# Feasibility of multiorgan risk prediction with routinely collected diagnostics: a prospective cohort study in the UK Biobank

**DOI:** 10.1136/bmjebm-2023-112518

**Published:** 2024-05-06

**Authors:** Celeste McCracken, Zahra Raisi-Estabragh, Liliana Szabo, Michele Veldsman, Betty Raman, Anya Topiwala, Adriana Roca-Fernández, Masud Husain, Steffen E Petersen, Stefan Neubauer, Thomas E Nichols

**Affiliations:** 1 Division of Cardiovascular Medicine, Radcliffe Department of Medicine, University of Oxford, and Oxford University Hospitals NHS Foundation Trust, Oxford, UK; 2 William Harvey Research Institute, Queen Mary University of London, London, UK; 3 Barts Heart Centre, St Bartholomew’s Hospital, Barts Health NHS Trust, London, UK; 4 Heart and Vascular Center, Semmelweis University, Budapest, Hungary; 5 Department of Experimental Psychology, University of Oxford, Oxford, UK; 6 Big Data Institute, Li Ka Shing Centre for Health Information and Discovery, Nuffield Department of Population Health, University of Oxford, Oxford, UK; 7 Perspectum Ltd, Oxford, UK; 8 Wellcome Centre for Integrative Neuroimaging (WIN FMRIB), University of Oxford, Oxford, UK; 9 Nuffield Department of Clinical Neuroscience, University of Oxford, Oxford, UK; 10 Health Data Research UK, London, UK; 11 Alan Turing Institute, London, UK

**Keywords:** PRIMARY CARE, General Practice, Primary Healthcare

## Abstract

**Objectives:**

Despite rising rates of multimorbidity, existing risk assessment tools are mostly limited to a single outcome of interest. This study tests the feasibility of producing multiple disease risk estimates with at least 70% discrimination (area under the receiver operating curve, AUROC) within the time and information constraints of the existing primary care health check framework.

**Design:**

Observational prospective cohort study

**Setting:**

UK Biobank.

**Participants:**

228 240 adults from the UK population.

**Interventions:**

None.

**Main outcome measures:**

Myocardial infarction, atrial fibrillation, heart failure, stroke, all-cause dementia, chronic kidney disease, fatty liver disease, alcoholic liver disease, liver cirrhosis and liver failure.

**Results:**

Using a set of predictors easily gathered at the standard primary care health check (such as the National Health Service Health Check), we demonstrate that it is feasible to simultaneously produce risk estimates for multiple disease outcomes with AUROC of 70% or greater. These predictors can be entered once into a single form and produce risk scores for stroke (AUROC 0.727, 95% CI 0.713 to 0.740), all-cause dementia (0.823, 95% CI 0.810 to 0.836), myocardial infarction (0.785, 95% CI 0.775 to 0.795), atrial fibrillation (0.777, 95% CI 0.768 to 0.785), heart failure (0.828, 95% CI 0.818 to 0.838), chronic kidney disease (0.774, 95% CI 0.765 to 0.783), fatty liver disease (0.766, 95% CI 0.753 to 0.779), alcoholic liver disease (0.864, 95% CI 0.835 to 0.894), liver cirrhosis (0.763, 95% CI 0.734 to 0.793) and liver failure (0.746, 95% CI 0.695 to 0.796).

**Conclusions:**

Easily collected diagnostics can be used to assess 10-year risk across multiple disease outcomes, without the need for specialist computing or invasive biomarkers. Such an approach could increase the utility of existing data and place multiorgan risk information at the fingertips of primary care providers, thus creating opportunities for longer-term multimorbidity prevention. Additional work is needed to validate whether these findings would hold in a larger, more representative cohort outside the UK Biobank.

**WHAT IS ALREADY KNOWN ON THIS TOPIC**

**WHAT THIS STUDY ADDS**

- In this study, we show that information already being collected as part of the primary care health check could feasibly be combined into a single calculator providing 10-year risk estimates for multiple diseases across related organ systems of heart, brain, liver and kidney. Moreover, much of the essential information can be acquired remotely.

**HOW THIS STUDY MIGHT AFFECT RESEARCH, PRACTICE OR POLICY**

- When patients attend their health check, they could potentially receive risk scores for multiple disease outcomes in addition to cardiovascular risk. Having earlier access to multiorgan information has the potential to enable earlier intervention for risk factors, more targeted use of resources and more effective multimorbidity prevention.

## Introduction

 Multimorbidity presents an urgent and increasing health challenge for ageing populations,^1^ with implications for health equity, disability and healthcare costs.^2 3^ Experts warn that effective handling of multimorbidity will require a multisystem approach^4^ prioritising proactive, rather than reactive, care,^5 6^ with primary care taking a leading role in chronic disease prevention.^7^,^9^

Assessment of atherosclerotic cardiovascular disease (CVD) risk is central to primary care and is now quick and easy due to widely available risk calculator tools such QRISK3^10^ and Framingham Risk Score.^11^ In the UK, primary care risk assessment has been codified in the form of the National Health Service (NHS) Health Check.^12 13^ At the health check, a number of clinical parameters are collected, CVD risk is assessed and the general practitioner directs personalised interventions that influence the long-term health trajectory of the patient. Despite urgent calls for more preventative attention to other diseases,^14 15^ there are currently no existing methods for multidisease risk prediction in primary care.

The primary targets of the NHS Health Check are heart disease, diabetes, stroke, dementia, kidney and liver disease, as laid out in the official guidance,^12^ website^16^ and patient information.^17^ These conditions are known to share underlying mechanisms^18^,^20^ and to co-occur in multimorbidity clusters.^21^,^23^

The objective of this study is to examine the feasibility of expanding the primary care health check to include risk assessment across multiple diseases. We focus on the 10 most commonly occurring serious conditions across the heart, brain, kidney and liver, namely, myocardial infarction, atrial fibrillation, heart failure, stroke, all-cause dementia, chronic kidney disease, fatty liver disease, alcoholic liver disease, liver cirrhosis and liver failure. Having access to a wider panel of risk information could lead to earlier disease detection, more targeted interventions and more effective prevention of longer-term multimorbidity.

However, there are several important challenges to consider. First, although risk scores have previously been developed for each additional condition (eg, dementia) there is simply not enough time within a 10–15 min consultation to gather all the required inputs and to calculate each risk score separately.^24^ A preferred solution would involve a single pool of inputs, and a single data entry page, from which multiple risk estimates could be calculated simultaneously.

Second, individual risk scores differ by the people they exclude, depending on the cohort in which they were developed.^25^ This leads to shifting sets of calculators (and required inputs) in the hands of the physician depending on the existing comorbidities of the patient. Instead, future solutions would include a person’s medical history and existing diagnoses, and adjust risk estimates accordingly.^26 27^

Third, not all health measures are equally accessible. NHS England is actively exploring ways that remote healthcare solutions can be used effectively to ease health service usage and make primary care services more accessible to all.^28^,^30^ These objectives call us to reflect on the information that is easily obtained and consider whether simple metrics can be potentially powerfully combined.

**Figure 1:**
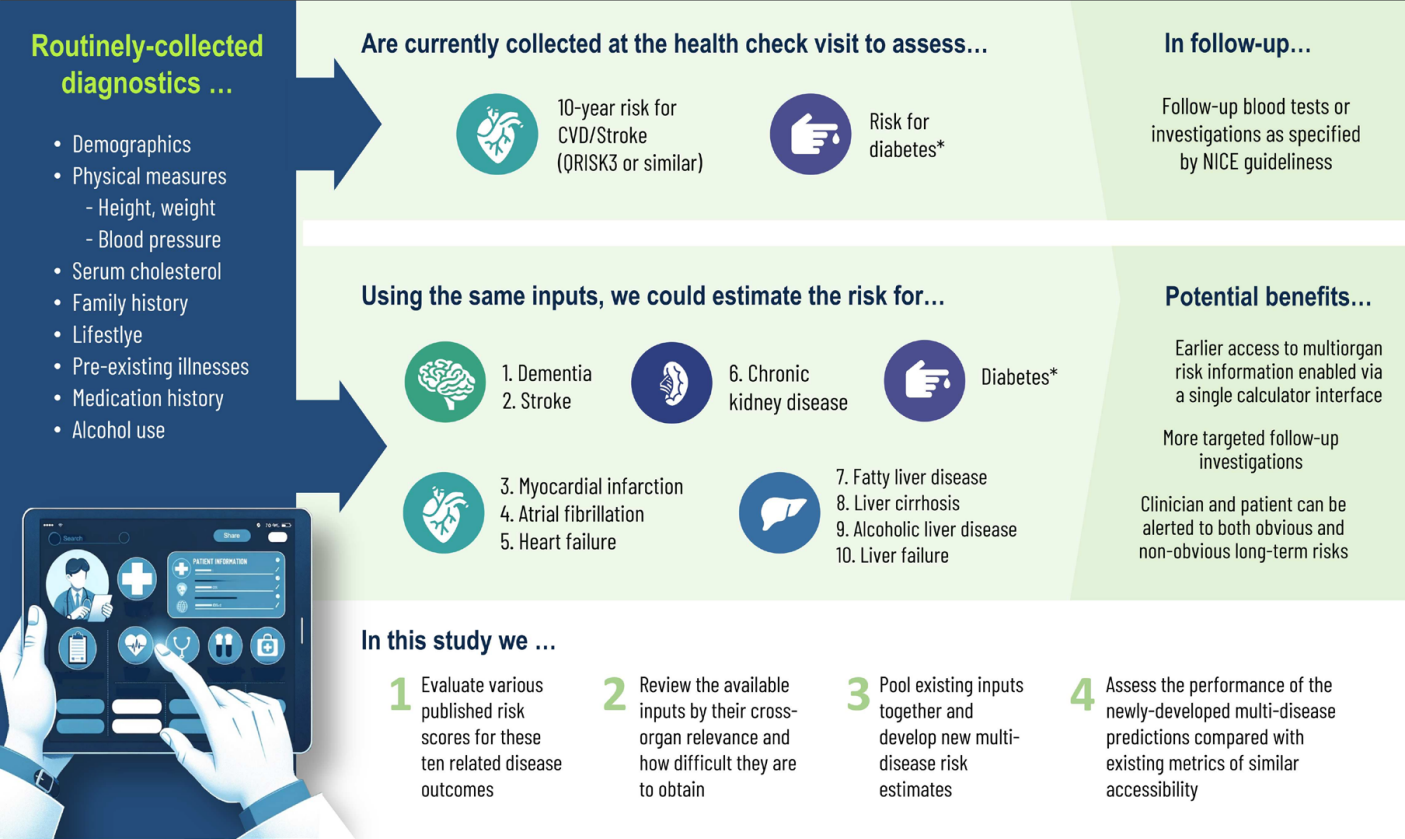
What can existing information tell us about multiorgan disease risk? In the context of increasing multimorbidity, we examine the feasibility of extending the existing primary care health check framework to include risk assessment for multiple diseases within the heart-brain-liver-kidney cluster. We evaluate a range of existing risk scores and consider whether and how they could be blended, and whether information already being collected could be effectively reused. If successful, this expansion could lead to earlier disease detection, more effective prevention and better resource allocation for multimorbidity prevention. *The diabetes screening component of the NHS Health Check protocol is not part of this analysis and would exist unchanged in both versions. CVD, cardiovascular disease; NICE, National Institute for Health and Care Excellence; NHS, National Health Service.

In this study, we use the UK Biobank data resource to emulate the information available within the primary care setting. Our objective is to explore the feasibility of multidisease risk estimation with easily collected diagnostics (figure 1), setting the minimum acceptable performance of 0.70 area under the receiver operating curve (AUROC) across all outcomes (as per Fagerland and, Hosmer, p177^31^). We begin by evaluating a range of published risk scores and assessing their performance in the UK Biobank cohort. We review the component inputs for each of those risk indices and identify scores that can be applied fully remotely (ie, without direct in-person contact) and those with a standard set of in-person inputs. Finally, we reuse the standard set of inputs to develop new risk scores compare their performance with existing risk scores.

## Methods

### Setting and study population

UK Biobank is a large prospective cohort study with participants drawn from the general population.^32^ UK residents aged 40–69 years old who are registered with a general practitioner, as identified from NHS registers, were invited to participate. Baseline data collection took place between 2006 and 2010, where registration date was used as the index date for the study. Follow-up events were ascertained via linked health records with latest censor date of 31 October 2022. Comprehensive details regarding linked primary care and hospital records are provided in official UK Biobank resources.^33 34^ To focus on 10-year risk estimation for all outcomes, follow-up was truncated at 10 years following baseline, giving a median follow-up time of 10 years (IQR=10–10). The clinical and demographic parameters collected in UK Biobank mimic those available in primary care and permit population-based modelling of multiorgan risk from baseline features.

From the overall UK Biobank cohort (n=502 386), 1298 participants were removed due to self-withdrawal or loss to follow-up, and 271 386 participants did not have primary care data available. There were 229 702 remaining participants who were confirmed to be present in both the main dataset and the primary care dataset. From these, a further 1462 participants were excluded due to missing values for height, weight, waist or hip circumference, leaving a final sample of 228 240 participants (see online supplemental figure 1A and online supplemental methods SM2).

### NHS Health Check and easily collected diagnostics

The NHS Health Check is a preventative primary care initiative (https://www.healthcheck.nhs.uk/)^12^ that forms the situational anchor for our study. Briefly, healthy people aged between 40 and 74 years are invited to visit their primary care team, where an inexpensive set of diagnostics are collected and 10-year risk of CVD is calculated using the widely validated QRISK3 calculator^10^ (https://qrisk.org/three/) or similar tool (figure 1).

Simple self-reported measures such as age, sex, family history, lifestyle factors, current medications and medical history are features that can be reported verbally and can be collected fully remotely (ie, without in-person contact). Physical measures such as height, weight, waist and hip circumference are easily measured without specialised technology. These are categorised as ‘remote features’ and are shown in the first two columns of online supplemental table 1. The term ‘remote’ is used to convey that remote collection of these parameters is possible, whether by phone or via an online form. Remote features can also be collected in person as part of the primary care visit.

Best practice guidelines^12^ specify a minimum set of parameters to be collected as part of the standard NHS Health Check protocol, consistent with the use of QRISK3. These include a number of remote features, with the addition of blood pressure measurement and blood tests for total and high-density lipoprotein (HDL) cholesterol. In this study, we include all remote features plus the required NHS Health Check parameters (blood pressure and cholesterol) under the category of ‘standard features’.

Finally, there are various blood/biochemistry tests that are widely available but are not part of the existing first-line health check protocol. These measures were identified based on their inclusion in existing research or risk scores (see ‘Existing risk scores’below) and have been included as an additional analysis to evaluate their potential incremental utility. These are shown in the fourth column of online supplemental table 1, and the full set of features including additional blood tests are referred to as ‘extended features’.

### Ascertainment of outcomes

Diagnoses and dates for the 10 disease outcomes (itemised above) were collated across multiple UK Biobank sources including self-report, linked hospital and primary care records and deaths, using published code lists where available.^35^,^37^ Incident outcomes were defined by first occurrence of disease after baseline recruitment. Participants with a record of the same disease at baseline were excluded from modelling for that disease, and follow-up was censored at either death or the study end date. In addition to the defined outcomes, a wide selection of other potentially relevant diagnoses was collected using the same multisource approach (see online supplemental table 1). A full listing of UK Biobank codes for outcomes ascertainment is provided in online supplemental table 2.

### Existing risk scores

**Figure 2:**
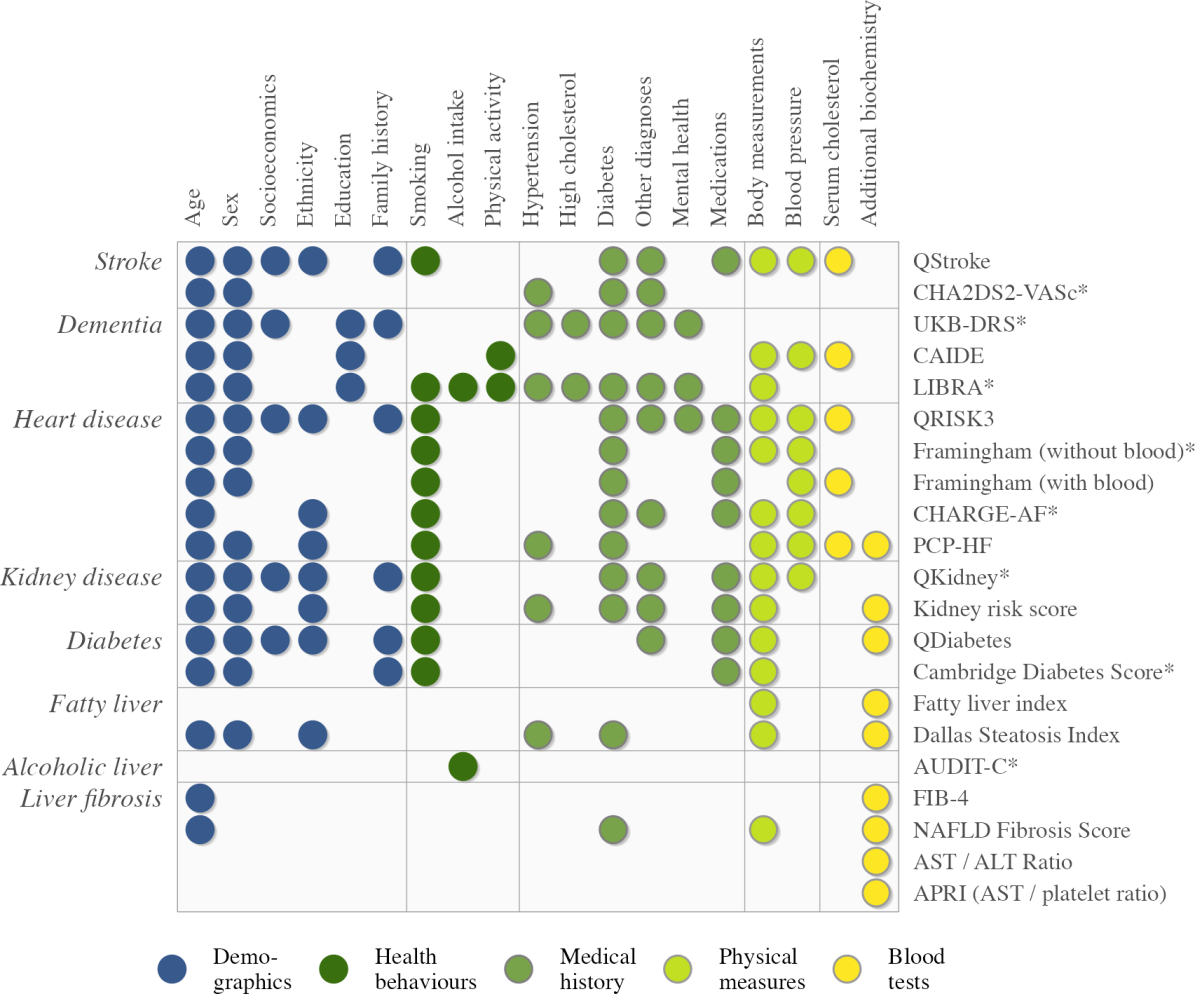
Risk scores across heart, brain, liver and kidney disease and their overlapping constituents. Coloured dots indicate measures that are included in the published risk scores shown on the right-hand side. Outcomes that are targeted by each risk score are shown on the left-hand side. Risk scores that can be implemented fully remotely are shown with an asterisk (*), in other words, risk scores that can be calculated without in-person contact. Physical measures include height, weight, waist circumference, hip circumference and resting heart rate. See online supplemental table 3 for a detailed listing of risk scores and their inputs. ALT, alanine aminotransferase; AST, aspartate aminotransferase; CAIDE, dementia risk score from the Cardiovascular Risk Factors, Aging and Dementia study; CHA2DS2-VASc, a score comprising congestive heart failure, hypertension, age, diabetes, prior stroke or transient ischaemic attack, vascular disease and sex; CHARGE-AF, Cohorts for Heart and Aging Research in Genomic Epidemiology model for atrial fibrillation; LIBRA, Lifestyle for Brain Health score; UKB-DRS, UK Biobank Dementia Risk Score.

We calculated QRISK3 for all study participants, along with 21 other published risk scores targeting disease risk across heart, brain, liver and kidney (see figure 2). These risk scores were selected based on a literature search for the most frequently used metrics for each outcome, the availability of published equations and the availability of online calculators for quality checking. Detailed information for all published indices is provided in online supplemental table 3. For incident stroke risk we considered QStroke^38^ and CHA2DS2-VASc,^39^ a score comprising congestive heart failure, hypertension, age, diabetes, prior stroke or transient ischaemic attack, vascular disease and sex. For all-cause dementia, we included three dementia risk scores; one developed using the CAIDE study (Cardiovascular Risk Factors, Aging and Dementia),^40^ the Lifestyle for Brain Health score (LIBRA)^41^ and the recently developed UK Biobank Dementia Risk Score (UKB-DRS).^37^ For myocardial infarction and heart failure, we considered Framingham Risk Score (with and without blood lipids),^11^ the Pooled Cohort Equations to Prevent Heart Failure (PCP-HF risk score ^42^) and QRISK3.^10^ For atrial fibrillation, we applied the Cohorts for Heart and Aging Research in Genomic Epidemiology model for atrial fibrillation (CHARGE-AF^43^). Chronic kidney disease risk (stage 3+) was predicted by two versions of QKidney^44^ and a Kidney Risk Score developed by Nelson and colleagues.^45^ For fatty liver disease, we considered the Fatty Liver Index^46^ and the Dallas Steatosis Index.^47^ Three diabetes risk scores (two versions of QDiabetes^48^ and Cambridge Diabetes Score^49^) were included as possibly useful predictors. AUDIT-C^50^ (the Alcohol Use Disorders Identification Test) is a questionnaire designed to assess risk for alcoholic liver disease, however, only the first AUDIT question was available in the UK Biobank with a high degree of data completeness. Lastly, liver fibrosis was represented by three candidate scores; the Fibrosis-4 Index (FIB-4^51^), the nonalcoholic fatty liver disease (NAFLD) fibrosis score^52^ and APRI (the aspartate aminotransferase/platelet ratio^53^). All published risk scores have been separately validated in their own studies. To assess their general utility, all risk scores were applied to the whole sample and to 10-year follow-up regardless of restrictions present in each respective derivation cohort.

### Existing risk scores inherit their type from their inputs

At the high level, examination of score inputs (figure 2) shows significant overlap in feature topics, however, the finer level detail (online supplemental table 3) reveals significant variability in input requirements. Moreover, the inputs to each risk score differ by accessibility, in other words, risk score inputs are often a mixture of remote and standard features, with occasional additional blood tests. Extending the health information framework described above, we categorise any risk score that is comprised only of inputs that can be collected remotely as a remote risk score. UKB-DRS^37^ (dementia) and Framingham risk score (using body mass index, BMI)^11^ are examples of this. With the most restricted set of inputs, we would expect these models to be the least powerful and have the lowest predictive performance. Risk scores that include remote and standard features are considered standard risk scores (eg, QRISK,^10^ QStroke^38^), while risk scores that require additional blood tests fall into the category of extended risk scores (eg, Nelson Kidney Risk Score,^45^ Fatty Liver Index^46^). We would expect risk models with access to the extended set of inputs (standard inputs plus additional biochemistry) to have the highest predictive performance. These categories are relevant to the process of building and comparing risk scores, such that performance comparisons are between models of the same type.

### Ascertainment of other features

Age at baseline, self-reported sex, systolic blood pressure, pulse rate and anthropomorphic measurements were taken at baseline, along with a touchscreen questionnaire collecting information about ethnicity, education, family history, smoking, alcohol use and physical activity. Townsend Deprivation Index at baseline was assigned based on participant postcode. Ethnic groups and smoking categories were recoded to match QRISK3 specifications. Education was coded as binary—‘Do you have any postsecondary/college/university qualifications?’ Physical activity was dichotomised to greater than or equal to 600 summed metabolic equivalent task minutes per week,^54^ approximately equivalent to 20 min exercise per day.^55^ Family history was drawn from self-reported illnesses of mother, father and siblings, where age of illness was not specified. Descriptive statistics and source information for study features are provided in online supplemental table 4. Blood sampling was carried out as part of the baseline assessment, providing measures of total cholesterol, HDL cholesterol and additional biochemistry (online supplemental table 5). Missingness among remote features was very small (<1%) while missingness among blood test variables ranged between 3% and 14%. Covariate missingness was handled with multiple imputation, with details provided in online supplemental methods SM3 and online supplemental table 6.

### Statistical analysis

Statistical analysis was with R V.4.1.2 and RStudio V.2022.02.0. We randomly stratified the data to create a training set (70%, 159 768 participants) and an internal validation set (30%, 68 472 participants) following the Strengthening the Reporting of Observational studies in Epidemiology (STROBE) checklist (online supplemental table 7). Standard checks confirmed that the characteristics of the training and validation cohorts were not significantly different.

#### Pairwise modelling of cross-disease associations

Prior to full-scale modelling, we sought to describe the associations between diseases. We examined the association of prevalent conditions with the risk of incident conditions using Cox proportional hazards regression, adjusting by age, sex, postsecondary education, ethnicity, smoking, physical activity, alcohol intake frequency, BMI, Townsend Deprivation Index, family history (of heart disease, stroke and dementia), any cancer diagnosis, hypertension, high cholesterol and diabetes. Proportionality was checked with visualisation of residuals across all models. This analysis was performed using the whole cohort (n=228 240). Due to the large number of tests in this section, all coefficient tests were adjusted for multiple testing via the Benjamini-Hochberg method^56^ with a false discovery rate of 5%.

#### Evaluation of existing risk scores

From the initial set of 22 published risk indices described above, we identified the best-performing score for each outcome across the 10 years of follow-up in the whole sample. Importantly, we evaluated all risk-score-outcome combinations, checking for potential predictive utility of each index beyond its original derivation cohort and intended outcome. We identified the risk score with highest discriminative performance, as measured by AUROC. AUROC was selected as the primary criterion for risk score performance because it does not depend on a specific prediction threshold and is more effective than simple accuracy in situations where rare events are being predicted.

#### New models for heart, brain, liver and kidney disease

Then, new models were developed for each of the 10 outcomes in the training set, and their performance for 10-year prediction was assessed in the internal validation set (online supplemental figure 1B). Model fitting was carried out (1) using remote features, (2) using standard features and (3) using the extended set of features. Crucially, at each level, we restricted all models (across the 10 related outcomes) to draw from the same pool of predictors. Feature selection was conducted using a stability selection approach,^57^ combining lasso Cox regression with bootstrapping to systematically identify the predictors that show consistent importance, thereby simplifying the final model and mitigating the risk of overfitting (see online supplemental methods SM4).

For each outcome, the final set of predictors was placed into a single survival model, with coefficients and prediction thresholds calculated using the training set, and predictions scored for discriminative accuracy in the validation set. Across all models, prediction performance was assessed with multiple metrics including AUROC, sensitivity, specificity, Somer’s Dxy and Brier score. Differences in performance metrics were further bootstrapped with 1000 bootstrapped samples to derive uncertainty estimates. To calculate sensitivity and specificity, the prediction thresholds were set to maximise balanced accuracy, given by (sensitivity+specificity)/2.^58^ Comparative performance was further evaluated with calibration plots to visually assess the alignment of predicted probabilities with actual outcomes, and reclassification statistics (integrated discrimination improvement (IDI) and continuous net reclassification improvement (cNRI)) to quantify any incremental improvements between existing and new models (online supplemental methods SM5 and SM6).

### Patient and public involvement

Patients and/or the public were not involved in the design, conduct, reporting or dissemination plans of this research.

## Results

### Participant characteristics

Overall, the study sample (n=228 240) was 45.3% male and 54.7% female, with an average age of 56.5 years at baseline (SD 8.1 years, table 1). Prevalence of hypertension, high cholesterol and diabetes at baseline was 32.6%, 20.8% and 5.5%, respectively. The most common incident event was atrial fibrillation (n=9997; 4.4%), and liver failure was the least common (n=340 events; 0.1%). Training and internal validation sets were similar across baseline variables and outcomes.

**Table 1:** Sample characteristics

Characteristic	Whole sample (n=228 240)	Training set (n=159 768)	Internal validation set (n=68 472)
Age (years)	56.5 (±8.1)	56.5 (±8.1)	56.5 (±8.1)
Sex: female	124 891 (54.7%)	87 563 (54.8%)	37 328 (54.5%)
Sex: male	103 349 (45.3%)	72 205 (45.2%)	31 144 (45.5%)
Townsend Deprivation Index	−1.34 (±3.03)	−1.34 (±3.03)	−1.35 (±3.03)
Postsecondary education			
No	91 042 (39.9%)	63 739 (39.9%)	27 303 (39.9%)
Yes	135 207 (59.2%)	94 625 (59.2%)	40 582 (59.3%)
(Missing)	1991 (0.9%)	1404 (0.9%)	587 (0.9%)
White ethnicity	217 863 (95.5%)	152 469 (95.4%)	65 394 (95.5%)
All other ethnicity groups	10 377 (4.5%)	7299 (4.6%)	3078 (4.5%)
Current smoker	23 934 (10.5%)	16 716 (10.5%)	7218 (10.5%)
Physically active (METS≥600)	169 005 (74.0%)	118 544 (74.2%)	50 461 (73.7%)
Alcohol intake less than once per week	70 489 (30.9%)	49 341 (30.9%)	21 148 (30.9%)
Alcohol intake once a week or more	157 751 (69.1%)	110 427 (69.1%)	47 324 (69.1%)
Self-reported health			
Excellent	36 350 (15.9%)	25 469 (15.9%)	10 881 (15.9%)
Good	131 466 (57.6%)	91 985 (57.6%)	39 481 (57.7%)
Fair	48 542 (21.3%)	33 949 (21.2%)	14 593 (21.3%)
Poor	10 634 (4.7%)	7475 (4.7%)	3159 (4.6%)
(Missing)	1248 (0.5%)	890 (0.6%)	358 (0.5%)
Physical measurements			
Body mass index (BMI kg/m^2^)	26.8(24.2, 30.0)	26.8(24.2, 30.0)	26.8(24.2, 30.0)
Obesity (BMI≥30 kg/m^2^)	57 469 (25.2%)	40 375 (25.3%)	17 094 (25.0%)
Waist circumference (cm)	90.3 (±13.5)	90.4 (±13.5)	90.3 (±13.5)
Resting heart rate (bpm)	69.4 (±11.3)	69.4 (±11.3)	69.4 (±11.2)
Systolic blood pressure (mm Hg)	138.2 (±18.7)	138.2 (±18.6)	138.2 (±18.7)
Total/HDL cholesterol ratio	4.14 (±1.13)	4.14 (±1.13)	4.14 (±1.13)
Risk factors			
Hypertension	74 503 (32.6%)	52 240 (32.7%)	22 263 (32.5%)
High cholesterol	47 471 (20.8%)	33 315 (20.9%)	14 156 (20.7%)
Diabetes	12 543 (5.5%)	8917 (5.6%)	3626 (5.3%)
Chronic obstructive pulmonary disease	5288 (2.3%)	3706 (2.3%)	1582 (2.3%)
Any cancer	21 896 (9.6%)	15 307 (9.6%)	6589 (9.6%)
Existing conditions at baseline			
Stroke	4284 (1.9%)	2994 (1.9%)	1290 (1.9%)
Alzheimer’s disease/dementia	346 (0.2%)	246 (0.2%)	100 (0.1%)
Myocardial infarction	6664 (2.9%)	4719 (3.0%)	1945 (2.8%)
Atrial fibrillation	4232 (1.9%)	2964 (1.9%)	1268 (1.9%)
Heart failure	1677 (0.7%)	1194 (0.7%)	483 (0.7%)
Chronic kidney disease	5610 (2.5%)	3886 (2.4%)	1724 (2.5%)
Fatty liver disease	1063 (0.5%)	718 (0.4%)	345 (0.5%)
Alcoholic liver disease	434 (0.2%)	298 (0.2%)	136 (0.2%)
Liver cirrhosis	498 (0.2%)	326 (0.2%)	172 (0.3%)
Liver failure	255 (0.1%)	170 (0.1%)	85 (0.1%)
Diagnoses after baseline			
Stroke	3953 (1.7%)	2728 (1.7%)	1225 (1.8%)
Dementia	2720 (1.2%)	1911 (1.2%)	809 (1.2%)
Myocardial infarction	5953 (2.6%)	4122 (2.6%)	1831 (2.7%)
Atrial fibrillation	9997 (4.4%)	6977 (4.4%)	3020 (4.4%)
Heart failure	4988 (2.2%)	3500 (2.2%)	1488 (2.2%)
Chronic kidney disease (stages 3, 4 and 5)	8698 (3.8%)	6116 (3.8%)	2582 (3.8%)
Fatty liver disease	3585 (1.6%)	2534 (1.6%)	1051 (1.5%)
Alcoholic liver disease	561 (0.2%)	402 (0.3%)	159 (0.2%)
Liver cirrhosis	951 (0.4%)	685 (0.4%)	266 (0.4%)
Liver failure	340 (0.1%)	236 (0.1%)	104 (0.2%)
Follow-up time (years, median, IQR)	10 (10–10)	10 (10–10)	10 (10–10)

Entries are either counts (percentages), mean (standard deviationSD) or median (25th percentile, 75th percentile). equivalent task, summed per week. index.

BMIbody mass indexHDLhigh-density lipoproteinMETSmetabolic equivalent task, summed per week

### Associations between existing disease and future disease risk

**Figure 3:**
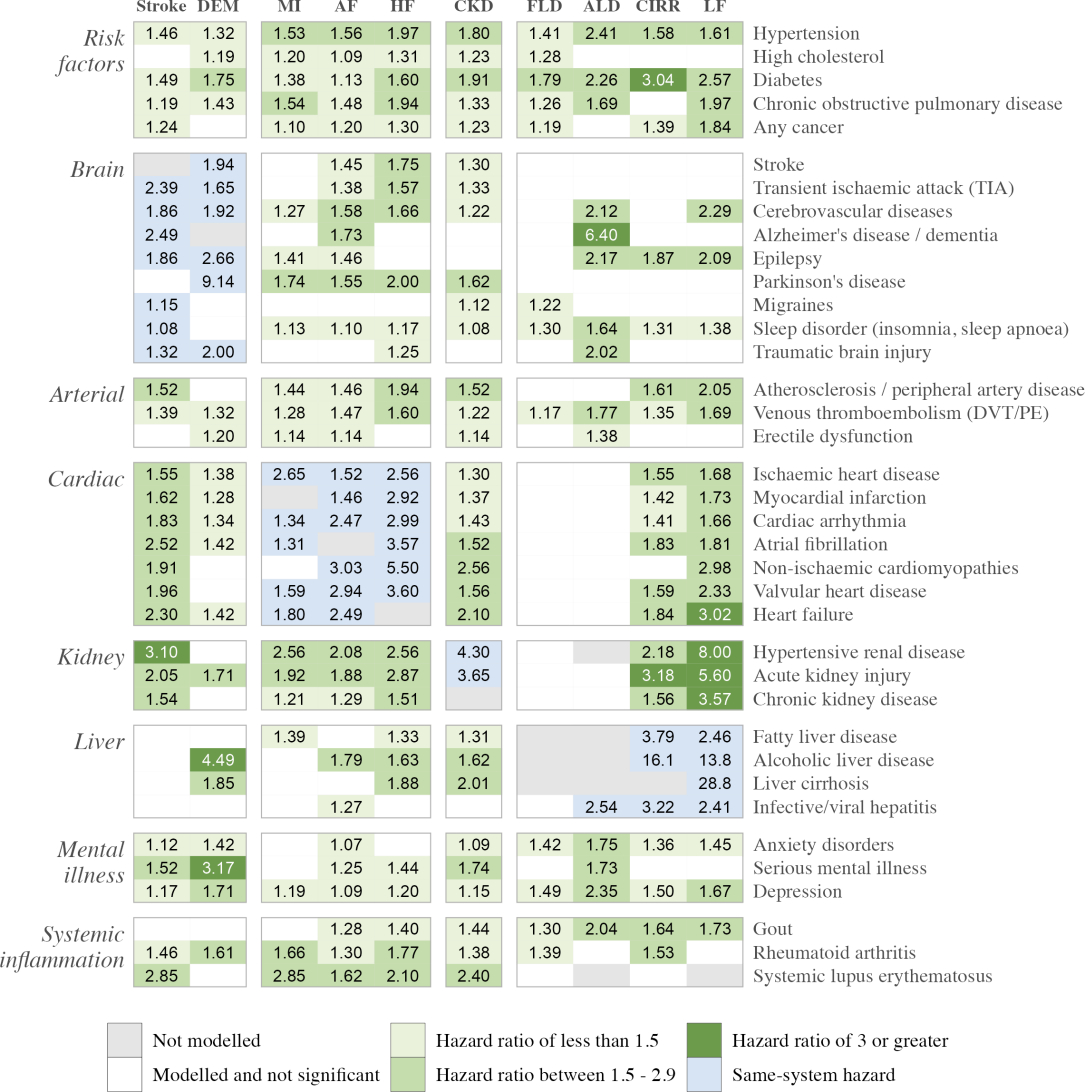
HRs for incident heart-brain-liver-kidney outcomes by existing disease diagnoses at baseline. Each entry shows the HR for incident outcomes (shown along the top) associated with the presence of existing risk factors, diagnoses and medication at baseline (shown down the right-hand side) in the whole cohort (n=228,240), using Cox-proportional hazards regression. For example, pre-existing hypertension increases the 10-year risk of stroke by 46%. Models are adjusted by age, sex, postsecondary education, ethnicity, smoking, physical activity, alcohol intake frequency, body mass index, Townsend Deprivation Index, family history (of heart disease, stroke and dementia), any cancer diagnosis, hypertension, high cholesterol and diabetes. HR significance was adjusted for multiple testing with a false discovery rate of 5%, where non-significant results are shown as empty white cells. Each result is from a different model. See online supplemental table 8 for detailed results. AF, atrial fibrillation; ALD, alcoholic liver disease; CKD, chronic kidney disease (stages 3, 4 or 5); CIRR, cirrhosis; DEM, all-cause dementia; DVT, deep vein thrombosis; FLD, fatty liver disease; HF, heart failure; LF, liver failure; MI, myocardial infarction; PE, pulmonary embolism.

Pairwise Cox analysis between heart-brain-liver-kidney outcomes and existing disease diagnoses revealed multiple cross-organ associations (figure 3, online supplemental table 8). All major heart diseases were significantly associated with increased risk of stroke, liver failure and the development of chronic kidney disease. All non-infective liver diseases at baseline were associated with increased risk of heart disease within 10 years, with an 88% increased risk of heart failure in participants with cirrhosis (HR 1.88, 95% CI 1.31 to 2.71, p=7.05×10^−4^), and a 39% increased risk of myocardial infarction in participants with fatty liver disease at baseline (1.39, 95% CI 1.08 to 1.80, p=0.012). Participants with alcoholic liver disease at baseline had a 4.5-fold risk for all-cause dementia (4.49, 95% CI 3.10 to 6.49, p=1.46×10^−15^) while rheumatoid arthritis at baseline conferred a 61% increased risk (1.61, 95% CI 1.30 to 1.99, p=1.50×10^−5^). Diagnosis of kidney or systemic inflammatory disease at baseline was associated with increased 10-year risk for disease across heart, brain and liver. Depression diagnosis at baseline had significant associations with disease across all four organs while serious mental illness (bipolar/schizophrenia/other psychosis) was associated with increased risk of alcoholic liver disease (HR 1.73, 95% CI 1.04 to 2.85, p=0.033), chronic kidney disease (HR 1.74, 95% CI 1.49 to 2.02, p=6.78×10^−13^) and all-cause dementia (HR 3.17, 95% CI 2.58 to 3.90, p=9.66×10^−28^).

### Best existing risk scores

**Figure 4:**
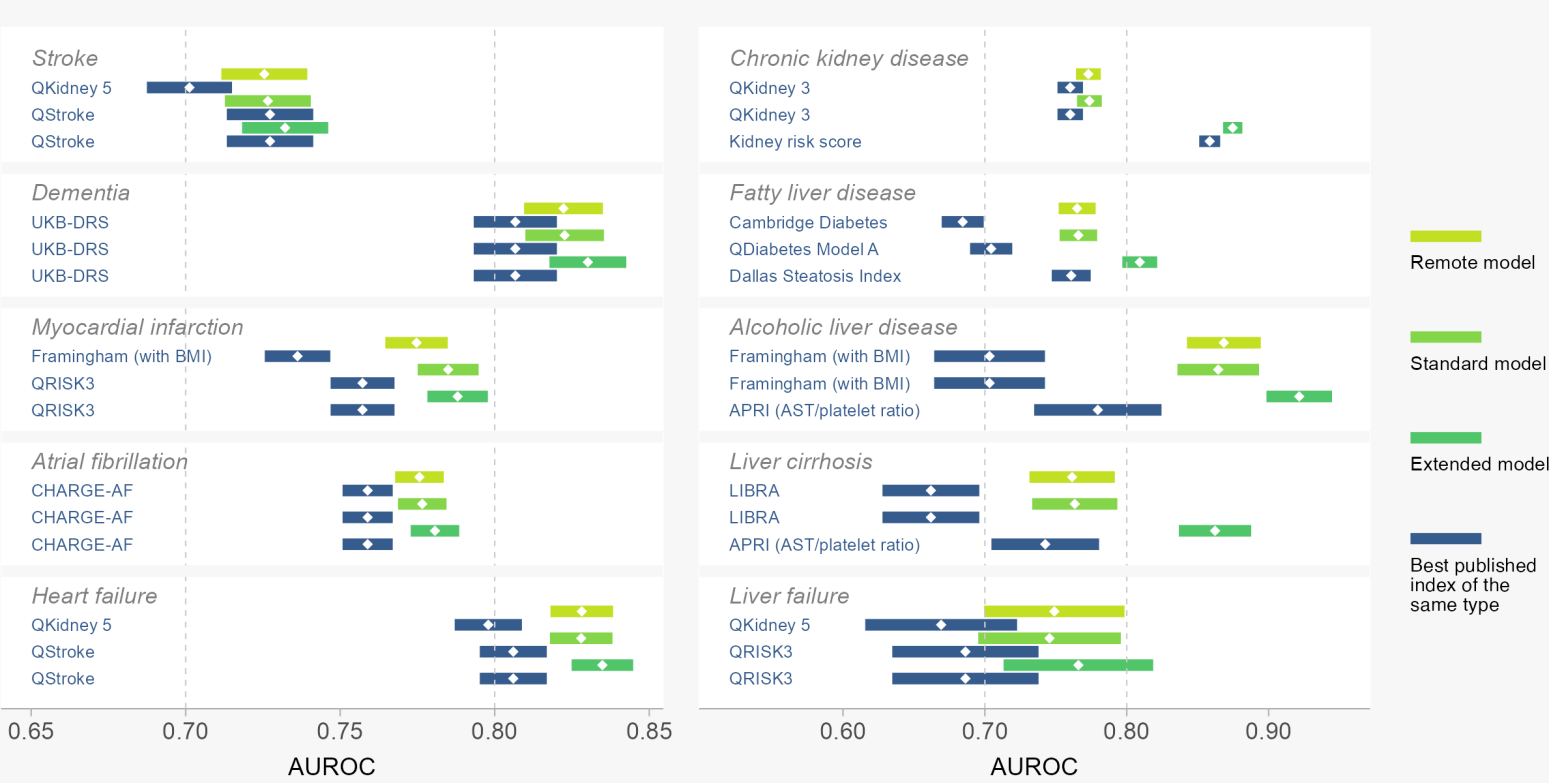
Performance of multiorgan risk scores in the validation set. Comparison of area under the receiver operating curve (AUROC, also known as concordance or C-statistic) for 10-year risk prediction across 10 outcomes. Horizontal bars show the 95% CI for validation set AUROC with uncertainty estimated from 1000 bootstrapped samples. Predictions from existing risk scores are shown in dark blue, while newly developed risk scores are shown in green. Remote models contain health metrics that can be answered verbally or self-measured easily by the patient. Standard models contain all remote metrics plus blood pressure and serum cholesterol tests. Extended models contain further blood tests. See online supplemental table 10 for detailed results. BMI, body mass index; UKB-DRS, UK Biobank Dementia Risk Score; APRI, the ratio of aspartate aminotransferase (AST) to platelet count.

Details of the three best-performing existing risk scores of each type (by highest sample AUROC) for each level of accessibility are shown in online supplemental table 9. From here, the one risk score with the highest AUROC was applied as the comparator in the validation sample, shown by dark blue bars in figure 4 with additional details in online supplemental table 10. At least one existing risk score surpassed the minimum adequate AUROC (0.70) for all outcomes except liver failure, with CHARGE-AF providing the best performance for atrial fibrillation across all levels (AUROC 0.759, 95% CI 0.751 to 0.767)), and UKB-DRS performing best for all-cause dementia (0.807, 95% CI 0.793 to 0.820)). Please note, that where a lower-level (remote or standard) model performs better than all more complex (extended) models for the same outcome, it will be retained as the best model at that level. Within the remote models, QKidney 5 had the highest AUROC for stroke (0.701 (95% CI 0.687 to 0.715)) and a surprisingly high remote model AUROC for heart failure (0.798 (95% CI 0.787 to 0.809)). Within standard models, we found several expected risk-score-outcome pairings, namely QStroke for stroke (0.727 (95% CI 0.714 to 0.741)), QRISK3 for myocardial infarction (0.757 (95% CI 0.747 to 0.767)) and QKidney three for chronic kidney disease (0.760 (95% CI 0.751 to 0.769)). Unexpectedly, QStroke also had the highest AUROC for 10-year heart failure (0.806 (95% CI 0.795 to 0.817)).

### Multiorgan risk prediction

The prediction performance of the newly developed risk scores is presented as green bars in figure 4 with additional details in online supplemental table 10. In general, the new risk score models performed as well or better than existing risk scores for all outcomes, with all new models achieving AUROC above 0.70. Using standard health check predictors, newly developed models performed significantly better than existing risk scores for some outcomes, with higher AUROC for myocardial infarction of 0.785 with 95% CI (0.775 to 0.795)), atrial fibrillation (0.777 (95% CI 0.768 to 0.785)), heart failure (0.828 (95% CI 0.818 to 0.838)), fatty liver disease (0.766 (95% CI 0.753 to 0.779)), alcoholic liver disease (0.864 (95% CI 0.835 to 0.894)) and liver cirrhosis (0.763 (95% CI 0.734 to 0.793)). Newly developed risk models had similar performance to existing scores for stroke (0.727 (95% CI 0.713 to 0.740)), dementia (0.823 (95% CI 0.810 to 0.836)) and chronic kidney disease (0.774 (95% CI 0.765 to 0.783)).

Importantly, for all outcomes studied, newly developed models using only remote features were able to achieve similar discriminative accuracy (AUROC) to their respective standard models.

When the set of additional biochemistry was added to the pool of potential predictors (in the extended models), there was very little incremental increase in predictive performance for heart and brain outcomes. In contrast, extended model features produced significantly better predictions for chronic kidney disease (AUROC 0.875, 95% CI 0.868 to 0.881), fatty liver (0.809, 95% CI 0.797 to 0.822), alcoholic liver (0.922, 95% CI 0.899 to 0.944) and cirrhosis (0.862, 95% CI 0.837 to 0.888). This improvement is substantial compared with existing risk scores and standard model estimates, suggesting that an approach with more blood biomarkers might be better at picking up these abnormalities. As expected, calibration statistics (online supplemental figure 3) and reclassification indices (online supplemental table 11) showed better calibration and significantly improved reclassification in the newly developed risk scores compared with existing risk scores, for example, standard myocardial infarction IDI=0.014, 95% CI (0.011 to 0.017) and cNRI=0.633, 95% CI (0.588 to 0.677). In nearly all comparisons, newly developed models had lower Brier scores (less average squared error) and higher Somers’ Dxy (better rank correlation) than existing models. While there were some instances of improved sensitivity in the newer models (stroke, myocardial infarction, chronic kidney disease, alcoholic liver and cirrhosis), overall, the improvements in discrimination were mainly driven by better specificity (fewer false positives, online supplemental table 10).

### The heart-brain-liver-kidney risk model coefficients

The large number of coefficients for multioutcome models are provided as beta coefficients in online supplemental spreadsheet 1, and as HRs in online supplemental spreedsheet 2, with a visual overview in online supplemental tables 4−6.

## Discussion

In this proof-of-concept analysis with 228 240 UK Biobank participants, we demonstrated that easily collected diagnostics can be used to assess risk across multiple disease outcomes. We have shown how this can be done without specialist computing or invasive biomarkers.

Pairwise modelling showed a complex pattern of cross-system associations, building on prior efforts to understand multimorbidity in the heart-brain-liver-kidney cluster.^23^ We confirmed that disease risk across all four organs was significantly associated with well-known risk factors such as hypertension, diabetes and high cholesterol; as well as other factors that have not yet been incorporated into standard risk paradigms beyond QRISK3, such as mental illness, systemic inflammation, sleep quality, arterial health and medication use.^59 60^

Recent studies have shown significant improvements in cardiovascular risk prediction using large data sets and machine learning methods.^60^,^63^ However, these studies still only target one organ (the heart), and when compared with conventional statistical models, deep learning or other ‘black box’ methods are not as readily explainable or easily translatable to clinical use.^64^ Several studies have tackled multidisease prediction. Bayati *et al*^65^ use multitask learning and group dimensionality reduction to identify a reduced pool of health check features to predict heart-brain-liver-kidney outcomes across 2 years follow-up. Most similar to the current work, Mahajan *et al*^66^ used electronic health records to derive multiple organ-specific risk scores, with a high degree of discrimination (AUROC>0.80 across heart, brain, lung, kidney and digestive disease). However, this study predicted hospital readmission using previous admissions for the same disease, whereas our models predict risk of new-onset disease.

The current study has several important limitations. We recognise the importance of thoroughly evaluating existing risk instruments before moving forward with new risk score development. We have begun this process, but there is more work to be done. On the other hand, by restricting the pool of input variables, new risk score development may well be required to meet this constraint, particularly where remote risk scores of adequate quality do not yet exist.

We acknowledge that the internal validation performance of our scores (developed within UK Biobank) is not directly comparable with external validation performance of published risk scores developed outside UK Biobank. Furthermore, each published risk score has a range of validation values across published work. For example, the validation AUROC provided by the original QRISK3 paper^10^ was 0.88 for women and 0.86 for men. Since then other external validation performance has varied in a numerical range consistent with the current work, with values of 0.707 for women and 0.681 for men reported for the 45–64 age group in the Clinical Practice Research Datalink,^67^ and values of 0.722 for women and 0.697 for men in a recent validation in UK Biobank.^68^

It may seem unconventional to apply existing risk score instruments outside their intended cohort (eg, including people with comorbidities) and outside their intended outcome (eg, using QRISK3 to predict myocardial infarction rather than combined CVD). Our study is not the first to explore this approach^25 69^ and to provide essential evidence for whether existing scores hold unrealised potential in additional contexts.

Although large compared with some, we caution that this study is small compared with larger risk score development projects, and we have not provided an external validation cohort. Furthermore, UK Biobank participants are subject to self-selection bias, and as such, they are known to be healthier and less ethnically diverse than the UK population.^70^ Therefore, any final models with this approach will require further recalibration and validation in large nationally representative cohorts. We acknowledge that there are some variables that are not well measured in the UK Biobank. Where these come up in multidisease risk equations, these are likely to be more accurately captured in a primary care-specific database.

In conclusion, this analysis demonstrates the feasibility of using standard health check predictors to produce multidisease risk estimates of reasonable quality. Such an approach has the potential to ease pressure on primary care, allowing physicians more time to focus on interpretation and follow-up^71^ thus providing new opportunities for multimorbidity prevention.

## supplementary material

10.1136/bmjebm-2023-112518 online supplemental file 1

10.1136/bmjebm-2023-112518 online supplemental file 2

10.1136/bmjebm-2023-112518 online supplemental file 3

## Data Availability

Data may be obtained from a third party and are not publicly available.
