# Supplementary material for: Feasibility of multiorgan risk prediction with routinely collected diagnostics: a prospective cohort study in the UK Biobank
Source: BMJ Evid Based Med. 2024 May 6;29(5):e112518. doi: 10.1136/bmjebm-2023-112518 (PMC11503151; doi:10.1136/bmjebm-2023-112518)
Supplement: online supplemental file 1 [file bmjebm-29-5-s001.pdf]

The feasibility of multi-organ risk prediction with routinely collected diagnostics

Table of Contents

Supplementary Figure 1: Sample selection and project workflow 2

Supplementary Methods 3
- SM1. Efforts to address potential sources of bias 3
- SM2: Sample size calculation 3
- SM3: Missing value handling 3
- SM4: Stability selection to identify final set of predictors 4
- SM5: Calibration 5
- SM6: Reclassification indices 5
- References for Supplementary Methods 5

Supplementary Table 1: Easily-collected features 7

Supplementary Table 2: Code definitions for outcome ascertainment 8

Supplementary Table 3: Selected published indices for heart-brain-liver-kidney risk 11

Supplementary Table 4: Covariate handling, descriptive statistics and source IDs 14

Supplementary Table 5: Biochemistry handling, descriptive statistics and source IDs 15

Supplementary Table 6: Missing value imputation details 16

Supplementary Table 7: STROBE Checklist 17

Supplementary Figure 2: Stability retention threshold evaluations 19

Supplementary Table 8: Details of pairwise Cox analysis 20

Supplementary Table 9: Performance of existing risk indices in the whole sample 23

Supplementary Table 10: Summary of fitted model performance by bootstrapping in the validation cohort. 25

Supplementary Figure 3: Calibration plots 27

Supplementary Table 11: Reclassification indices 28

Supplementary Figure 4: Hazard ratios for Remote model coefficients 29

Supplementary Figure 5: Hazard ratios for Standard model coefficients 30

Supplementary Figure 6: Hazard ratios for Extended model coefficients 31

Supplementary Figure 1: Sample selection and project workflow

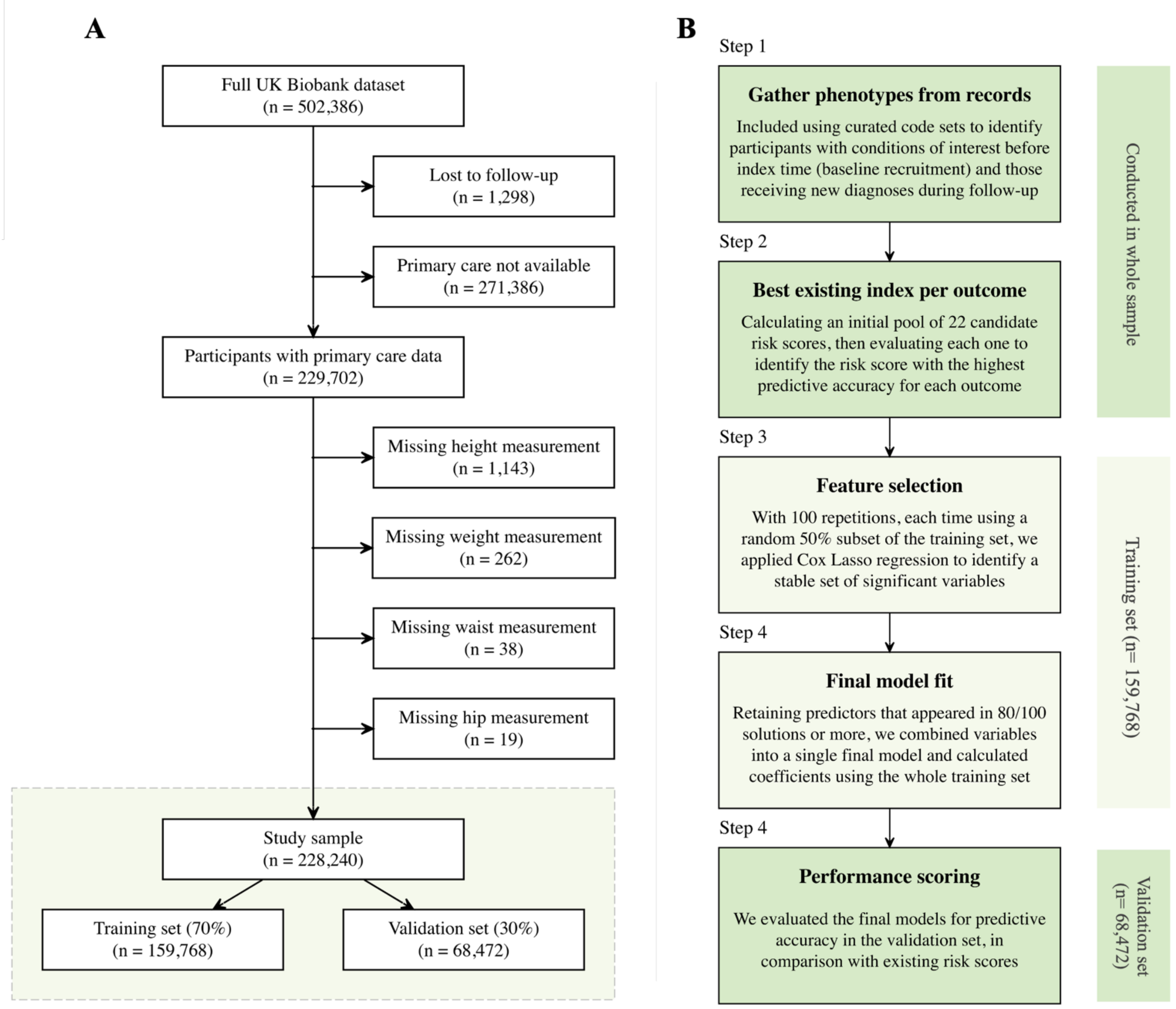

Panel A provides the sample selection flow diagram, while Panel B provides a workflow of the analyses.

Supplementary Methods

SM1. Efforts to address potential sources of bias

To minimise potential selection bias, invitations for UK Biobank recruitment were sent to a random sample of the UK population that were registered with NHS general practitioners. However, due to the voluntary nature of participation in the UK Biobank prospective cohort, self-selection bias remains in the sample. As mentioned in the discussion of the paper, UK Biobank participants are known to be healthier and less ethnically diverse than the UK population[1].Therefore, any final models with this approach will require further recalibration and validation in large nationally-representative cohorts.

To minimise information bias, all baseline data were collected using standardised instruments and procedures. Data collectors underwent standardised training to ensure consistent and accurate data collection. To minimise measurement bias from clinical outcome ascertainment, outcomes were taken from linked death records, hospital records and primary care records. In other words, self-reported health conditions were used to ascertain diagnosis status prior to the index date (baseline recruitment) but were not relied upon as evidence of outcome events. Codes for disease outcome definitions were developed lists using published code lists where available[2–4].

To minimise confounding bias, we identified a wide pool of potential predictors a priori based on existing literature and subject matter expertise. These confounders were included in our statistical models as candidate predictors.

A very small number of participants were lost to follow-up (1,298 participants, equivalent to 0.5% of the final sample). On average, those who were lost to follow-up were slightly younger and healthier (with existing diagnoses across heart, brain, liver and kidney) but were more likely to have non-white ethnicity and higher socioeconomic deprivation.

SM2: Sample size calculation

The overall aim of sample selection for this study was to identify the largest cohort possible that had both primary care available and complete cases across a core set of covariates (age, sex, height, weight, waist and hip measurement). From the initial cohort of 502,386 UK Biobank participants, 1,298 participants were excluded due to self-withdrawal or loss to follow-up. Of the remaining participants, 229,702 participants had primary care data available, evidenced by registered consent the presence of their patient ID in the primary care data set). From these, a further 1,462 participants were excluded due to missing values for height (n= 1,143), weight (n=262), waist (n=38) and hip circumference (n=19), leaving a final sample of size of 228,240 participants. A study selection diagram and overall workflow is provided in Supplementary Figure 1A.

SM3: Missing value handling

All missing value handling has the same aim, in other words, to allow the inclusion of the (majority) non-missing row of data into the analysis, without introducing any new bias with the value that is imputed. In analysis with many input variables, scattered missingness can have a substantial deleterious effect on sample size when one must remove the whole row for each missing value. Numerical details of missing value imputation are provided in Supplementary Table 6.

Our internal standard protocol is to always filter to complete cases across age, sex, height and weight. Next, in our data, socioeconomic deprivation has a unimodal continuous distribution, a very small rate of missingness, and its value cannot reasonably be estimated from other variables. Therefore, we

replaced these with the mean so that the results of any analysis including imputed values will not be significantly different from analysis without them.

For the categorical ethnicity variable, there is again a very small degree of missingness, which cannot be predicted from age, sex and body size, and has a very large majority category (95.5% "White"). Therefore, we assigned these values to the overwhelming majority group to have a much smaller chance of introducing bias than assigning them to the minority category.

Finally, the remaining set of variables are continuous, have slightly larger rates of missingness, and are health metrics that contain some native associations between them, and associations with our foundational variables of age, sex, BMI and waist-to-hip ratio. Therefore, for this set, multiple imputation with chained equations (MICE) was the ideal method to produce imputed values that reflect the underlying pattern of correlations and allow the use of all present data whilst introducing the least possible amount of bias. The MICE algorithm makes iterative predictions of the missing value given all other predictors and then reviews the overall variation introduced by imputation. This is refined over 20 iterations until we arrive at a set of imputations for missing values that sit very nicely within the correlation context of the overall. We used the 'mice' package in R to complete this procedure[5]

SM4: Stability selection to identify final set of predictors

In this study, we had a large pool of predictors to choose from. In the remote model phase, there were p1= 93 predictors to choose from, for standard features p2= 97, and for extended features p3= 129. The aim was to obtain the best model fit possible, with the simplest model possible (a small number of predictors) and minimise the risk of overfitting. In this case, overfitting occurs when the final models are so tightly tuned to the UK Biobank dataset that they perform poorly on any other dataset. Stability selection[6]is a method developed to identify features that consistently contribute to the predictive power of the model across various subsets of the data, and to penalise the inclusion of non-informative variables that could otherwise lead to overfitting.

The process begins by creating 100 bootstrapped samples of 50% of the training set. A bootstrapped sample is a random sample with replacement, meaning that some members of the sample might be included more than once. For this study, the training set has n=159,768, so we drew 100 bootstrapped samples of size nsub= 79,884.

Each subset is then subjected to a feature selection process that incorporates modelling with regularisation to favour sparser models, thereby implicitly penalising the selection of superfluous features. In this study, within each bootstrapped sample, we fit lasso regression, with an underlying Cox model, using 5-fold cross-validation to tune for the local minimum lambda. In this way, each subsample produces its own penalised (simplified) Cox model, with only a reduced set of predictors chosen out of the total pool.

The essence of stability selection lies in the subsequent aggregation phase, where the selection frequencies of features across all subsamples are compiled. This frequency data serves as the basis for determining the stability of each feature's selection, highlighting those consistently deemed important.

A critical step in the stability selection method is the establishment of a frequency threshold to discern stable features. Features surpassing this predetermined threshold are considered sufficiently stable and are thus selected for inclusion in the final model. For example, from our 100 subsample Cox models, should we include only predictors that were consistently retained in all 100, or maybe look for predictors that were featured in 90/100 models? This thresholding decision is pivotal and adjustable, reflecting a trade-off between the desire to encompass a broader array of features and the risk of incorporating irrelevant ones.

In this study, we evaluated model fit and parsimony across stability retention thresholds between 50% and 100%. We found that using a conservative 80% cut-off resulted in models with a much smaller set

of predictors, but with predictive accuracy (AUROC) that was not significantly different from the much larger better-fitting models. These model fit statistics are provided visually in Supplementary Figure 2. A diagram of the stability selection workflow is given in Supplementary Figure 1B, and although the overall pipeline was coded manually in R, the ‘glmnet’ package[7] in R was used to fit lasso Cox models and the ‘rsample’ package[8] was used for bootstrapping. A further example of stability selection implementation is provided by Haftorn and colleagues[9]

SM5: Calibration

Calibration plots are an essential tool for assessing the reliability of predictive models in binary classification tasks, providing insight into the agreement between predicted probabilities and observed outcomes. By plotting the predicted probability of an event (on the x-axis) against the observed occurrences of that event (on the y-axis), these plots reveal the model's accuracy in estimating real-world probabilities. This is particularly vital in medical decision-making, where the precision of probability estimates can significantly impact clinical outcomes and patient care strategies. A perfectly calibrated model would result in a plot closely adhering to the 45-degree line, indicating that the model's predictions are well-aligned with actual frequencies. In this study, the calibration plots were produced using the ‘predtools’ package[10] in R.

SM6: Reclassification indices

Reclassification indices, such as IDI (Integrated Discrimination Improvement) and NRI (Net Reclassification Improvement), are statistical measures used in the field of predictive modelling, particularly in the context of risk prediction models. They help assess small improvements in predictive performance when comparing two or more models or risk factors[11,12].

Integrated Discrimination Improvement (IDI) measures the improvement in the discrimination slope between a reference model and a new model. It is defined by the difference in the discrimination slopes (the difference between the average predicted probabilities of the events occurring versus non-occurring) between the new and the reference model. The IDI is a continuous measure that does not require predefined risk categories and can capture improvements in model performance even if the improvements do not change the risk category of individuals.

According to Steyerberg and colleagues, the IDI coefficient can be thought of as the difference in discrimination slopes of the two models, or the improvement in Pearson R-squared/2 or the decrease in scaled Brier score[13]

Continuous Net Reclassification Improvement (NRI) quantifies how well a new model assigns risk to subjects compared to an existing model. It is calculated by examining changes in the predicted risk (e.g., low, intermediate, high risk) and assesses whether individuals who experienced events (e.g., disease occurrence) are appropriately moved to higher risk values, and those who did not experience events are moved to lower risk values in the new model, when compared to the reference model.

Pencina and colleagues[12] stress that continuous NRI has no native interpretation in terms of percent of the sample or percentage improved, but that NRI is designed to quantify improvement in performance and hence its magnitude is more important than statistical significance.

References for Supplementary Methods

1 Huang JY. Representativeness Is Not Representative: Addressing Major Inferential Threats in the UK Biobank and Other Big Data Repositories. Epidemiology. 2021;189–93.

2 UK Biobank. Code lists for health outcomes. 2020. https://biobank.ndph.ox.ac.uk/showcase/refer.cgi?id=594 (accessed 23 March 2022)

3 Hayward KL, Johnson AL, Horsfall LU, et al. Detecting non-alcoholic fatty liver disease and risk factors in health databases: accuracy and limitations of the ICD-10-AM. BMJ Open Gastroenterology. 2021;8:e000572.

4 Anatürk M, Patel R, Ebmeier KP, et al. Development and validation of a dementia risk score in the UK Biobank and Whitehall II cohorts. BMJ Ment Health. 2023;26. doi: 10.1136/bmjment-2023-300719

5 van Buuren S, Groothuis-Oudshoorn K. mice: Multivariate imputation by chained equations in R. Journal of Statistical Software. 2011;45:1–67.

6 Meinshausen N, Bühlmann P. Stability selection. Journal of the Royal Statistical Society: Series B (Statistical Methodology). 2010;72:417–73.

7 Friedman J, Hastie T, Tibshirani R. Regularization Paths for Generalized Linear Models via Coordinate Descent. J Stat Softw. 2010;33:1–22.

8 Frick H. rsample 1.2.0 (R package documentation): General resampling infrastructure. 2023. https://cran.r-project.org/web/packages/rsample/rsample.pdf (accessed 2 February 2024)

9 Haftorn KL, Romanowska J, Lee Y, et al. Stability selection enhances feature selection and enables accurate prediction of gestational age using only five DNA methylation sites. Clinical Epigenetics. 2023;15:114.

10 Sadatsafavi M, Saha-Chaudhuri P, Petkau J. Model-based ROC (mROC) curve: examining the effect of case-mix and model calibration on the ROC plot. Med Decis Making. 2022;42:487–99.

11 Pencina MJ, D’ Agostino Sr RB, D’ Agostino Jr RB, et al. Evaluating the added predictive ability of a new marker: From area under the ROC curve to reclassification and beyond. Statistics in Medicine. 2008;27:157–72.

12 Pencina MJ, D’Agostino Sr RB, Steyerberg EW. Extensions of net reclassification improvement calculations to measure usefulness of new biomarkers. Statistics in Medicine. 2011;30:11–21.

13 Steyerberg EW, Vickers AJ, Cook NR, et al. Assessing the Performance of Prediction Models: A Framework for Traditional and Novel Measures. Epidemiology. 2010;21:128–38.

Supplementary Table 1: Easily-collected features

	**1. Remote features***		**2. Standard features**	**3. Extended features**
	**Features expected as part of the NHS Health Check**	**Features that could be added with minimal additional cost**	**In-person metrics that are specified as part of NHS Health Check**	**Features that are extra but may help detect / predict disease**
**Demographics**	Age, sex, ethnicity, postcode[1]	Self-reported education level		
**Family history**	Family history of heart disease	Family history of dementia, diabetes, or stroke		
**Lifestyle**	Smoking, alcohol use<br>General self-reported health	Physical activity, household occupancy (living alone)	All remote features plus …	Both other categories plus…
**Physical measures**	BMI (height, weight),	Waist-hip ratio (waist, hip in cm), resting pulse rate	SBP, DBP<br>SBP variability[2]	
**Blood/ biochemistry**			Total cholesterol, HDL cholesterol	Blood glucose, HbA1c, RBC, platelet count, CRP, GGT, AST, ALT, ALP, cystatin-C, serum creatinine, albumin, bilirubin, total protein, urate, urine creatinine
**Existing diagnoses / medical history**				
**Vascular/general risk factors**	Diabetes, hypertension, high cholesterol	Dizziness, fainting, shortness of breath, COPD, any cancer		
**Brain**	[Stroke, transient ischaemic attack][3]<br>Migraine	Other cerebrovascular disease, dementia (any cause), epilepsy, Parkinson's disease, multiple sclerosis, traumatic brain injury,<br>sleep disorder		
**Arterial**	Erectile dysfunction	Atherosclerosis, peripheral artery disease, venous thromboembolism		
**Heart**	[Coronary heart disease including angina and myocardial infarction][3]<br>Atrial fibrillation (AF)	Other (non-AF) cardiac arrhythmias, valvular heart disease, non-ischaemic cardiomyopathies, heart failure		
**Kidney**	Chronic kidney disease	Acute kidney injury, kidney stones		
**Liver**		NAFLD, hepatitis, alcoholic liver disease, cirrhosis		
**Systemic inflammation**	Rheumatoid arthritis, systemic lupus erythematosus	Gout		
**Mental health**	Depression, bipolar disorder, schizophrenia	Anxiety		
**Medications**	Anti-hypertensive medication, atypical antipsychotics, regular steroid tablets	Insulin, statins		

*The term "remote" features is used to convey that remote collection of these parameters is possible, whether by phone or via an online form. Remote features can also be collected in person as part of the primary care visit.1 Townsend deprivation score via postcode was dropped from the set of predictors, as this feature is difficult to implement in a simple portable spreadsheet and applies only to the UK. [2.] SBP variability was dropped from the set of predictors, as this feature is difficult to obtain in a single in-person visit. [3] These are diagnoses that the primary care physician would typically already be aware of, and would preclude attendance at an NHS Health Check. *Abbreviations:* BMI= body mass index, SBP = systolic blood pressure, NAFLD= non-alcoholic fatty liver disease, HDL= high-density lipoprotein, HbA1c = glycated haemoglobin, RBC= red blood cell count, CRP = C-reactive protein, GGT = gamma glutamyl transferase, AST = aspartate aminotransferase, ALT = alanine aminotransferase.

Supplementary Table 2: Code definitions for outcome ascertainment

Outcome / Source	UK Biobank Field ID or code	Evidence of
**Stroke**		
Self-report (20002)	1081, 1086, 1491, 1583	Past events
First occurrences	131360, 131362, 131366, 131368	Past events
Diagnosed by doctor (6150, 6152)	3, 4056	Past events
Algorithm	42006	Past and incident
ICD10	I60, I61, I63, I64	Past and incident
ICD9	430, 431, 434, 436	Past and incident
Primary care	G60.., G600., G601., G602., G603., G604., G605., G606., G60X., G60z., G61.., G610., G611., G612., G613., G614., G615., G616., G617., G618., G619., G61X., G61X0, G61X1, G61z., G63y0, G63y1, G64.., G640., G6400, G641., G6410, G64z., G64z0, G64z1, G64z2, G64z3, G64z4, G65z1, G66.., G660., G661., G662., G663., G664., G665., G666., G667., G668., G669., G6760, G6W.., G6X.., Gyu60, Gyu61, Gyu62, Gyu63, Gyu64, Gyu6E, Gyu6F, Gyu6G, X00D3, X00D4, X00D5, X00D6, X00D7, X00D8, X00D9, X00DA, X00DD, X00DE, X00Df, X00DF, X00Dg, X00DG, X00DI, X00DJ, X00DK, X00DM, X00DN, X00DO, X00DP, X00DQ, X00DR, X00DS, X00DT, X204F, Xa00I, Xa00J, Xa00K, Xa01b, Xa01c, Xa01h, Xa01i, Xa01j, Xa01k, Xa01l, Xa01m, Xa01o, Xa0Bj, Xa0kZ, Xa0N3, Xa0NJ, Xa0NK, Xa0NN, Xa0NO, Xa0NQ, Xa0NS, Xa0NV, Xa0NW, Xa0NX, Xa1hE, XaB4Z, XaBE2, XaBEC, XaBED, XaJgQ, XE0VJ, XE2aB	Past and incident events
**Dementia (any cause)**		
Self-report (20002)	1263	Past events
First occurrences	130836, 130838, 130840, 130842, 131036, 131038	Past events
Algorithm	42018, 42020, 42022, 42024	Past and incident
ICD10	F00, F01, F02, F03, F051, G30, G31	Past and incident
ICD9	290, 331	Past and incident
Primary care	.1461, .3AE3, .3AE4, .3AE5, .3AE6, .66h., .6AB., .9hD1, .9Ou., .9Ou1, .9Ou2, .9Ou3, .9Ou4, .9Ou5, .E11., .E111, .E112, .E113, .E114, .E115, .E116, .E11Z, .F21Z, .F371, .G78., 1461, 2233, 38C13, 3AE3., 3AE4., 3AE5., 3AE6., 66h.., 6AB.., 8BM02, 8BM50, 8BM60, 8BPa., 8CMe0, 8CMG2, 8CMZ., 8CMZ0, 8CMZ1, 8CMZ2, 8CMZ3, 8CSA., 8Hla., 8IAe0, 8IAe2, 9hD.., 9hD0., 9hD1., 9Ou., 9Ou.., 9Ou1., 9Ou2., 9Ou3., 9Ou4., 9Ou5., A411., A4110, E00.., E000., E001., E0010, E0011, E0012, E0013, E001z, E002., E0020, E0021, E002z, E003., E004., E0040, E0041, E0042, E0043, E004z, E012., E02y1, E0300, E0301, E0302, E0303, E0304, E0310, E0311, E0312, E0313, E0314, E041., Eu00., Eu000, Eu001, Eu002, Eu00z, Eu01., Eu010, Eu011, Eu012, Eu013, Eu01y, Eu01z, Eu02., Eu020, Eu021, Eu022, Eu023, Eu024, Eu025, Eu02y, Eu02z, Eu04., Eu040, Eu041, Eu04y, Eu04z, Eu106, Eu107, F1..., F10.., F10y., F10y0, F10y1, F10y2, F10yz, F10z., F11.., F110., F1100, F1101, F111., F112., F116., F118., F11x0, F11x2, F11x7, F11x9, F11xz, F11y., F11y2, F11yz, F11z., F1440, F21y2, Fyu30, Fyu31, Ub1T6, X002m, X002U, X002V, X002w, X002W, X002x, X002y, X002z, X0030, X0031, X0032, X0033, X0034, X0035, X0036, X0037, X0039, X003A, X003B, X003C, X003D, X003E, X003F, X003G, X003H, X003I, X003J, X003l, X003m, X003P, X003R, X003T, X003V, X003W, X003X, X004B, X004E, X005K, X005L, X005M, X005N, X005O, X005P, X00R0, X00R2, X00RI, X00RJ, X00Rk, X77qx, Xa0fZ, Xa0lH, Xa0sC, Xa0sE, Xa1GB, Xa25J, Xa3ez, Xa7nD, XaA1S, XaaBZ, XaaeA, XaaiW, Xabd2, Xabd3, XabEk, XabEl, XabtQ, XabVp, XacIx, XacIy, XacIz, XacJ0, XacLx, Xacly, Xaclz, XacM2, XaE74, Xaefu, XaIKB, XaIKC, XaIRJ, XaJBQ, XaJBU, XaJBV, XaJBW, XaJBX, XaJPy, XaKyY, XaLFf, XaLFo, XaLFp, XaMFy, XaMG0, XaMGF, XaMGG, XaMGI, XaMGJ, XaMGK, XaMJC, XaOfZ, XaPws, XaYFR, XaYPX, XaZqJ, XaZWz, XE15F, XE17j, XE1aG, XE1Xr, XE1Xs, XE1Xu, XE1Z6, XE1Z7, XE1Z8	Past and incident events
**Myocardial infarction**		
Self-report (20002)	1075	Past events
Diagnosed by doctor (6150, 6152)	1, 3894	Past events
First occurrences	131298, 131300, 131302	Past events
Algorithm	42000	Past and incident
ICD10	I21, I22, I23, I241, I252	Past and incident
ICD9	410, 411, 412, 429	Past and incident
Primary care	14A3., 14A4., 322.., 3222, 889A.00, G30.., G300., G301., G3010, G3011, G301z, G302., G303., G304., G305., G306., G307., G3070, G3071, G308., G309., G30A., G30B., G30X., G30X0, G30y., G30y0, G30y1, G30y2, G30yz, G30z., G31.., G310., G3110, G3115, G312., G31y., G31y0, G31y1, G31y2, G31y3, G31yz, G32.., G34.., G340., G3400, G3401, G341., G3410, G3411, G3412, G3413, G341z, G342., G343., G344., G34y., G34y0, G34y1, G34yz, G34z., G34z0, G35.., G350., G351., G353., G35X., G36.., G360., G361., G362., G363., G364., G365., G366., G38.., G380., G381., G382., G383., G384., G38z., G3y.., G3z.., G501., G501.00, G5y2., G704., G72B1, Gyu31, Gyu32, Gyu33, Gyu34, Gyu35, Gyu36, X2006, X200a, X200d,	Past and incident events

	X200D, X200e, X200G, X200H, X200I, X200J, X200K, X200L, X200M, X200N, X200O, X200P, X200Q, X200R, X200S, X200T, X200U, X200V, X200W, X200x, X200X, X200Y, X200Z, X201u, X2021, X202q, X202r, X203e, X204f, X204q, X77vP, X782C, X782F, Xa0YL, Xa6Yx, XaAC3, XaAzi, XaBL1, XaEgZ, XaeVd, XaFsH, XaG1Q, XaIf1, XaINF, XaIwM, XaIwY, XaJX0, XC0bX, XE0Uk, XE0WG, XE2aA, XM0rN, XM1Qk, XSDT6	
**Atrial fibrillation**		
Self-report (20002)	1471	Past events
First occurrences	131350	Past events
ICD10	I48, I480, I481, I482, I489	Past and incident
ICD9	4273	Past and incident
Primary care	.14AN, .662S, .6A9., .8666, .G67., .G670, 14AN., 662S., 6A9.., 7936A, G573., G5730, G5731, G5732, G5733, G5734, G5735, G5736, G5737, G5738, G5739, G573z, X202R, X202S, Xa2E8, Xa3rp, Xa7nI, XaaUH, XaDv6, XaEga, XaeUP, XaeUQ, XaeUR, Xafis, XaIIT, XaMGD, XaOfa, XaOft, XE0Wk, Y7820	Past and incident events
**Heart failure**		
Self-report (20002)	1076	Past events
First occurrences	131354	Past events
ICD10	I500, I501, I509	Past and incident
ICD9	4280, 4281, 4289	Past and incident
Primary care	.14A6, .14AM, .1O1., .662p, .662T, .662W, .8H2S, .8HBE, .G6A., .G6A1, .G6A2, .G6AZ, 14A6., 14AM., 1O1.., 662p., 662T., 662W., 8H2S., 8HBE., G1yz1, G232., G234., G58.., G580., G5800, G5801, G5802, G5803, G5804, G581., G5810, G582., G583., G584., G58z., G5y4z, SP111, X102Y, X202k, X202l, XaBwi, XaEgY, XafeB, XaIIU, XaIpn, XaIQM, XaIQN, XaKNW, XaLon, XaO5n, XaWyi, XaZIC, XE0V8, XE0V9, XE0Wo, XE2QG, Y27da, Y2b96	Past and incident events
**Chronic kidney disease**		
Self-report (20002)	1193	Past events
Self-report (20004)	1195, 1580, 1581, 1582	Past events
Algorithm	42026	Past and incident
ICD10	N165, N180, N183, N184, N185, N188, N189, T824, T861, Y602, Y612, Y622, Y841, Z490, Z491, Z492, Z940, Z992	Past and incident
ICD9	585, 5859	Past and incident
OPCS4	L741, L742, L743, L744, L745, L746, L748, L749, M012, M013, M014, M015, M018, M019, M023, M084, M172, M174, M178, M179, X401, X402, X403, X404, X405, X406, X407, X408, X409, X411, X412, X418, X419, X421, X428, X429, X431	Past and incident
Primary care	.1Z12, .1Z13, .1Z14, .1Z15, .1Z16, .1Z1B, .1Z1C, .1Z1D, .1Z1E, .1Z1F, .1Z1G, .1Z1H, .1Z1J, .1Z1K, .1Z1L, 1Z12., 1Z13., 1Z14., 1Z15., 1Z16., 1Z1a., 1Z1b., 1Z1B., 1Z1c., 1Z1C., 1Z1d., 1Z1D., 1Z1e., 1Z1E., 1Z1f., 1Z1F., 1Z1G., 1Z1H., 1Z1J., 1Z1K., 1Z1L., 1Z1T., 1Z1V., 1Z1W., 1Z1X., 1Z1Y., 1Z1Z., K05.., K050., K053., K054., K055., K0D.., X30In, X30J0, X30J1, XacAb, XacAd, XacAe, XacAf, XacAh, XacAi, XacAM, XacAN, XacAO, XacAV, XacAW, XacAX, XaLHI, XaLHJ, XaLHK, XaNbn, XaNbo, XaO3t, XaO3u, XaO3v, XaO3w, XaO3x, XaO3y, XaO3z, XaO40, XaO41, XaO42, XE0df	Past and incident events
**Fatty liver disease**		
First occurrences	131670	Past events
ICD10	K758, K760	Past and incident
Primary care	EMISR4QFA1, J61y1, J61y7, J61y8, J61y9, X307v, XaQIT	Past and incident
**Alcoholic liver disease**		
First occurrences	131658	Past events
ICD10	K70	Past and incident
ICD9	5710, 5711, 5712, 5713	Past and incident
Primary care	.I72., .I722, .I723, G8523, J610., J611., J612., J6120, J613., J6130, J617., J6170, X306r, X3071, X3072, X3073, XaBE3, XaC1d, XE0b4, XE0dD, XE0dF	Past and incident events
**Liver cirrhosis**		
Self-report (20002)	1141, 1158	Past events
First occurrences	131666	Past events
ICD10	I850, I859, I864, I982, I983, K740, K741, K742, K746, K766, K767, R18	Past and incident
ICD9	4560, 4561, 5715, 5722, 5723, 5724, 7895	Past and incident
Primary care	.I725, 2485, G85.., G850., G851., G852., G8520, G8521, G8522, G852z, G853., G857., G858., Gyu94, HNG0090, J61.., J615., J6150, J6151, J6152, J6153, J6154, J6155, J6156, J6157, J6158, J6159, J615A, J615B, J615C, J615D, J615E, J615F, J615G, J615H, J615y, J615z, J616., J6160, J6161, J6162, J616z, J61y., J61y1, J61y3, J61y4, J61y5, J61y6, J6356, Jyu71, X307L, X307M, X307N, X307O, X307P, X307S, X307T, X307U, X307V, Xa9C7, XaBM6, XE0b5, XE0b7, XE0bA, XE2up	Past and incident events
**Liver failure**		
Self-report (20002)	1158	Past events

First occurrences	131662	Past events
ICD10	K72, T864, Z944	Past and incident
ICD9	570	Past and incident
OPCS4	J01, X43	Past and incident
Primary care	.79L8, .I71., 7800, 78000, 78001, 7L1f., 7L1fy, 7L1fz, J60.., J600., J6000, J6001, J6002, J600z, J601., J6010, J6011, J6012, J601z, J60z., J6130, J61y0, J622., J625., J62y., SP086, SP142, X0042, X0058, X20a8, X3073, X3076, X3077, X3078, X3079, X307A, X307C, X307x, X307z, Xa8Df, XaMuG, XaMuH, XaMuI, XE0dB, ZV427	Past and incident events

ICD10 codes are drawn from fields 41270, 41280, 41234 and 41259; ICD9 codes are drawn from fields 41271, 41281, 41234 and 41259; OPCS4 codes are drawn from fields 41272, 41282, 41149 and 41259; Primary care codes are drawn from field 42040. Deaths codes are drawn from fields 40000, 40001 and 40023. Where a 3-digit code is given, this includes all 4-digit sub-codes, for example, I46 includes I462, I468 and I469.

Supplementary Table 3: Selected published indices for heart-brain-liver-kidney risk

Risk score	Target disease	References	Score type	Component inputs
**QStroke**	Stroke	Hippisley-Cox et al. (2013)(1)	Standard	Age, sex, UK postcode / Townsend deprivation index, ethnicity, smoking, BMI, SBP, family history: heart disease, total/HDL cholesterol ratio, diabetes, diabetes, ischaemic heart disease, atrial fibrillation, valvular heart disease, heart failure, chronic kidney disease, antihypertensive medication, rheumatoid arthritis
**CHA2DS2-VASc**	Stroke	Ntaios et al. (2013)(2)	Remote	Age, sex, diagnoses or history of heart failure, hypertension, diabetes, stroke, transient ischaemic attack, venous thromboembolism, myocardial infarction, peripheral artery disease
**QRISK3**	Atherosclerotic CVD (myocardial infarction, stroke or CVD death)	Hippisley-Cox et al. (2017a)(3)	Standard	Age, sex, UK postcode / Townsend deprivation index, ethnicity, smoking, BMI, SBP, SBP standard deviation, family history: heart disease, total/HDL cholesterol ratio, diabetes, diabetes, erectile dysfunction, migraines, atrial fibrillation, chronic kidney disease, atypical antipsychotic medication, antihypertensive medication, regular steroid tablets, rheumatoid arthritis, systemic lupus erythematosus, severe mental illness
**Framingham Risk Score (2 types)**	Atherosclerotic CVD	D'Agostino et al. (2008)(4)	Remote<br>Standard	Age, sex, current smoker, BMI, SBP, diabetes, antihypertensive medication (total cholesterol, HDL cholesterol)
**PCP-HF**	Heart failure	Khan et al. (2019)(5)	Extended	Age, sex, ethnicity, BMI, SBP, smoking, hypertension, diabetes, blood glucose, total cholesterol, HDL cholesterol
**UKB-DRS**	All-cause dementia	Anatürk et al. (2020)(6)	Remote	Age, sex, education, family history of dementia, hypertension, diabetes, high cholesterol, stroke, depression, elevated socioeconomic deprivation, lives alone at home.
**CAIDE**	All-cause dementia	Kivipelto et al. (2006)(7)	Standard	Age, sex, education, physical activity, BMI, SBP, total cholesterol
**LIBRA**	All-cause dementia	Schiepers et al. (2018)(8)	Remote	Age, sex, education, current smoker, alcohol intake frequency, physical activity, BMI, hypertension, high cholesterol, diabetes, ischaemic heart disease, depression
**CHARGE-AF**	Atrial fibrillation	Alonso et al. (2013)(9)	Remote	Age, ethnicity, current smoker, height, weight, DBP, SBP, diabetes, myocardial infarction, heart failure, antihypertensive medication
**QKidney (2 versions)**	Chronic kidney disease	Hippisley-Cox et al. (2010)(10)	Remote	Age, sex, UK postcode / Townsend deprivation index, ethnicity, smoking, BMI, SBP, family history: kidney disease, diabetes, diabetes, atherosclerosis / peripheral artery disease, heart disease, heart failure, kidney stones, antihypertensive medication, NSAID use, rheumatoid arthritis, systemic lupus erythematosus
**Kidney risk score**	Chronic kidney disease	Nelson et al. (2019)(11)	Extended	Age, sex, ethnicity, smoking, BMI, hypertension, diabetes, heart disease, insulin use, estimated glomerular filtration rate, glycated haemoglobin (hba1c), serum creatinine, urine albumin, urine creatinine

**Fatty liver index**	NAFLD	Bedogni et al. (2006)(12)	Extended	BMI, waist circumference, GGT, triglycerides
**Dallas Steatosis Index**	NAFLD	McHenry et al. (2020)(13)	Extended	Age, sex, ethnicity, BMI, hypertension, diabetes, alanine aminotransferase, glucose, triglycerides
**QDiabetes (2 versions)**	Undiagnosed current	Hippisley-Cox et al (2017b)(14)	Extended	Age, sex, ethnicity, UK postcode / Townsend deprivation index, BMI, smoking, atypical antipsychotic medication, regular steroid tablets, history of heart attack, angina, stroke or TIA, history of gestational diabetes (yes/no), learning difficulties, serious mental illness, polycystic ovarian syndrome, statins, antihypertensive medication, family history of diabetes. [HbA1c, blood glucose]
**Cambridge Diabetes**	Type 2 diabetes	Griffin et al. (2000)(15)	Remote	Age, sex, smoking, BMI, family history of diabetes, antihypertensive medication, prescribed steroids
**AUDIT-C**	Alcohol use disorder	Bush et al. (1998)(16)	Remote	Ten-item questionnaire about alcohol use. [Please note only the first question could be implemented in our sample due to high missingness]
**FIB-4**	Liver fibrosis /cirrhosis	Vallet-Pichard et al. (2007)(17)	Extended	Age, alanine aminotransferase, aspartate aminotransferase, platelet count
**NAFLD Fibrosis Score**	Liver fibrosis /cirrhosis	Angulo et al. (2007)(18)	Extended	Age, BMI, diabetes, albumin, alanine aminotransferase, aspartate aminotransferase, platelet count
**AST/Platelet ratio (APRI)**	Liver fibrosis /cirrhosis	Wai et al. (2003)(19)	Extended	Aspartate aminotransferase, platelet count
**ALBI (albumin/bilirubin)**	Severity grading in liver cancer	Johnson et al (2015)(20)	Extended	Bilirubin, albumin

References for Supplementary Table 3

1. Hippisley-Cox J, Coupland C, Brindle P. Derivation and validation of QStroke score for predicting risk of ischaemic stroke in primary care and comparison with other risk scores: a prospective open cohort study. BMJ. 2013 May 2;346:f2573.

2. Ntaios G, Lip GYH, Makaritsis K, Papavasileiou V, Vemmou A, Koroboki E, et al. CHADS2, CHA2DS2-VASc, and long-term stroke outcome in patients without atrial fibrillation. Neurology. 2013 Mar 12;80(11):1009–17.

3. Hippisley-Cox J, Coupland C, Brindle P. Development and validation of QRISK3 risk prediction algorithms to estimate future risk of cardiovascular disease: Prospective cohort study. BMJ Online. 2017 May 23;357(May):1–21.

4. D'Agostino RB, Vasan RS, Pencina MJ, Wolf PA, Cobain M, Massaro JM, et al. General Cardiovascular Risk Profile for Use in Primary Care. Circulation. 2008 Feb 12;117(6):743–53.

5. Khan SS, Ning H, Shah SJ, Yancy CW, Carnethon M, Berry JD, et al. 10-Year Risk Equations for Incident Heart Failure in the General Population. J Am Coll Cardiol. 2019 May;73(19):2388–97.

6. Anatürk M, Patel R, Ebmeier KP, Georgiopoulos G, Newby D, Topiwala A, et al. Development and validation of a dementia risk score in the UK Biobank and Whitehall II cohorts. BMJ Ment Health [Internet]. 2023 Jul 1 [cited 2023 Nov 13];26(1). Available from: https://mentalhealth.bmj.com/content/26/1/e300719

7. Kivipelto M, Ngandu T, Laatikainen T, Winblad B, Soininen H, Tuomilehto J. Risk score for the prediction of dementia risk in 20 years among middle aged people: a longitudinal, population-based study. Lancet Neurol. 2006 Sep 1;5(9):735–41.

8. Schiepers OJG, Köhler S, Deckers K, Irving K, O'Donnell CA, van den Akker M, et al. Lifestyle for Brain Health (LIBRA): a new model for dementia prevention. Int J Geriatr Psychiatry. 2018 Jan 1;33(1):167–75.

9. Alonso A, Krijthe BP, Aspelund T, Stepas KA, Pencina MJ, Moser CB, et al. Simple risk model predicts incidence of atrial fibrillation in a racially and geographically diverse population: the CHARGE-AF consortium. J Am Heart Assoc. 2013 Mar 18;2(2):e000102.

10. Hippisley-Cox J, Coupland C. Predicting the risk of Chronic Kidney Disease in Men and Women in England and Wales: prospective derivation and external validation of the QKidney®Scores. BMC Fam Pract. 2010 Jun 21;11(1):49.

11. Nelson RG, Grams ME, Ballew SH, Sang Y, Azizi F, Chadban SJ, et al. Development of Risk Prediction Equations for Incident Chronic Kidney Disease. JAMA. 2019 Dec 3;322(21):2104–14.

12. Bedogni G, Bellentani S, Miglioli L, Masutti F, Passalacqua M, Castiglione A, et al. The fatty liver index: A simple and accurate predictor of hepatic steatosis in the general population. BMC Gastroenterol. 2006 Nov 2;6(1):33.

13. McHenry S, Park Y, Browning JD, Sayuk G, Davidson NO. Dallas Steatosis Index Identifies Patients With Nonalcoholic Fatty Liver Disease. Clin Gastroenterol Hepatol. 2020 Aug 1;18(9):2073-2080.e7.

14. Hippisley-Cox J, Coupland C. Development and validation of QDiabetes-2018 risk prediction algorithm to estimate future risk of type 2 diabetes: cohort study. BMJ. 2017 Nov 20;359:j5019.

15. Griffin SJ, Little PS, Hales CN, Kinmonth AL, Wareham NJ. Diabetes risk score: towards earlier detection of Type 2 diabetes in general practice. Diabetes Metab Res Rev. 2000;16(3):164–71.

16. Bush K, Kivlahan DR, McDonell MB, Fihn SD, Bradley KA. The AUDIT Alcohol Consumption Questions (AUDIT-C): An Effective Brief Screening Test for Problem Drinking. Arch Intern Med. 1998 Sep 14;158(16):1789–95.

17. Vallet-Pichard A, Mallet V, Nalpas B, Verkarre V, Nalpas A, Dhalluin-Venier V, et al. FIB-4: An inexpensive and accurate marker of fibrosis in HCV infection. comparison with liver biopsy and fibrotest. Hepatology. 2007 Jul 1;46(1):32–6.

18. Angulo P, Hui JM, Marchesini G, Bugianesi E, George J, Farrell GC, et al. The NAFLD fibrosis score: A noninvasive system that identifies liver fibrosis in patients with NAFLD. Hepatology. 2007 Apr 1;45(4):846–54.

19. Wai CT, Greenson JK, Fontana RJ, Kalbfleisch JD, Marrero JA, Conjeevaram HS, et al. A simple noninvasive index can predict both significant fibrosis and cirrhosis in patients with chronic hepatitis C. Hepatology. 2003 Aug 1;38(2):518–26.

20. Johnson P j, Berhane S, Kagebayashi C, Satomura S, Teng M, Hl R, et al. Assessment of liver function in patients with hepatocellular carcinoma: a new evidence-based approach-the ALBI grade. J Clin Oncol Off J Am Soc Clin Oncol [Internet]. 2015 Feb 20 [cited 2023 Nov 21];33(6). Available from: https://pubmed.ncbi.nlm.nih.gov/25512453/

Supplementary Table 4: Covariate handling, descriptive statistics and source IDs

Category	Variable	Positive	Percent	Mean	SD	Min	25%	50%	75%	Max	N missing	% missing	UKB Field ID
Demographics	All rows	228,240	100%										
	Age (years)	-	0%	56.52	8.07	38	50	58	63	71	0	0.00%	21003
	Sex: Male	103,349	45%	0.45	0.5	0	0	0	1	1	0	0.00%	31
	Townsend deprivation index	-	0%	-1.34	3.03	-6.26	-3.64	-2.15	0.46	11	335	0.15%	189
	Post-secondary education (yes/no)	136,186	60%	0.6	0.49	0	0	1	1	1	1991	0.87%	6138
	Non-white ethnicity (yes/no)	10,377	5%	0.05	0.21	0	0	0	0	1	882	0.39%	21000
Lifestyle	Current smoker (yes/no)	23,934	10%	0.1	0.31	0	0	0	0	1	303	0.13%	20116
	Ex-smoker	78,512	34%	0.34	0.48	0	0	0	1	1	303	0.13%	
	Light smoker <10 per day	10,505	5%	0.05	0.21	0	0	0	0	1	303	0.13%	3456
	Moderate smoker (10 - 19 per day)	7,208	3%	0.03	0.17	0	0	0	0	1	303	0.13%	
	Heavy smoker (>= 20 per day)	6,280	3%	0.03	0.16	0	0	0	0	1	303	0.13%	
	Physical activity (summed MET-minutes per week)	-	0%	2370.47	2643.56	0	578	1493	3213	19278	1498	0.66%	
	Active (METS ≥ 600)	169,005	74%	0.74	0.44	0	0	1	1	1	1498	0.66%	
	Alcohol intake frequency: Times per month	-	0%	11.06	10.5	0	2	6	14	30	406	0.18%	1558
	Alcohol intake daily or nearly daily	44,943	20%	0.2	0.4	0	0	0	0	1	406	0.18%	
	Alcohol intake once a week or more	157,751	69%	0.69	0.46	0	0	1	1	1	406	0.18%	
	Alcohol: Non-drinker	18,631	8%	0.08	0.27	0	0	0	0	1	406	0.18%	
	Health: Excellent	36,458	16%	0.16	0.37	0	0	0	0	1	1248	0.55%	2178
	Health: Poor	10,757	5%	0.05	0.21	0	0	0	0	1	1248	0.55%	
	Lives alone at home	43,037	19%	0.19	0.39	0	0	0	0	1	1668	0.73%	709
Physical measures	Height (m)	-	0%	1.68	0.09	1.22	1.61	1.68	1.75	2.05	0	0.00%	50
	Weight (kg)	-	0%	78.18	15.97	33	66.6	76.5	87.7	197.7	0	0.00%	21002
	Hip circumference (cm)	-	0%	103.49	9.24	39	97	102	108	195	0	0.00%	49
	Waist circumference	-	0%	90.34	13.49	20	80	90	99	197	0	0.00%	48
	BMI: Underweight	1,123	0%	0	0.07	0	0	0	0	1	0	0.00%	
	BMI: Overweight	97,221	43%	0.43	0.49	0	0	0	1	1	0	0.00%	
	BMI: Obese 1 (30-35)	41,097	18%	0.18	0.38	0	0	0	0	1	0	0.00%	
	BMI: Obese 2 (> 35)	16,372	7%	0.07	0.26	0	0	0	0	1	0	0.00%	
	Waist-hip ratio	-	0%	0.87	0.09	0.2	0.8	0.87	0.94	2.13	0	0.00%	
	Pulse rate (bpm)	-	0%	69.4	11.27	30.5	61.5	68.5	76	173	233	0.10%	102, 95
	Pulse rate < 60	43,419	19%	0.19	0.39	0	0	0	0	1	233	0.10%	
	Pulse rate > 90	35,602	16%	0.16	0.36	0	0	0	0	1	233	0.10%	
	Diastolic blood pressure (DBP)	-	0%	82.44	10.15	32	75.5	82	89	147.5	233	0.10%	4079, 94
	Systolic blood pressure (SBP)	-	0%	138.19	18.67	72	125	136.5	150	253	235	0.10%	4080, 93
	SBP standard deviation	-	0%	5.48	4.49	0	2.12	4.24	7.78	84.85	7517	3.29%	
Family history	Family history: Heart disease (yes/no)	142,563	62%	0.62	0.48	0	0	1	1	1	0	0.00%	20110
	Family history: Dementia (yes/no)	27,072	12%	0.12	0.32	0	0	0	0	1	0	0.00%	20107
	Family history: Diabetes (yes/no)	55,756	24%	0.24	0.43	0	0	0	0	1	0	0.00%	20111
	Family history: Stroke (yes/no)	67,127	29%	0.29	0.46	0	0	0	1	1	0	0.00%	

Supplementary Table 5: Biochemistry handling, descriptive statistics and source IDs

Category	Variable	Mean	SD	Min	25%	50%	75%	Max	UKB Field ID	N missing	% missing
Standard biochemistry	Total cholesterol (mmol/L)	5.70	1.15	1.43	4.91	5.66	6.44	15.46	30690	13,153	5.8%
	HDL cholesterol (mmol/L)	1.45	0.38	0.22	1.17	1.40	1.67	4.40	30760	31,961	14.0%
Additional biochemistry	Alanine aminotransferase (U/L)	23.67	14.27	3.1	15.49	20.22	27.53	495.19	30620	13,233	5.8%
	Albumin (g/L)	45.19	2.63	20.67	43.47	45.17	46.9	59.46	30600	31,839	13.9%
	Alkaline phosphatase (U/L)	83.82	26.3	8.0	67.4	80.5	96.1	1416.7	30610	13,139	5.8%
	Aspartate aminotransferase (U/L)	26.25	10.47	3.3	21	24.4	28.9	500	30650	13,958	6.1%
	C-reactive protein (mg/L)	2.61	4.39	0.08	0.66	1.34	2.78	79.49	30710	13,606	6.0%
	Creatinine (enzymatic) in urine (micromole/L)	8897.91	5823.49	88	4383	7564	12092	60000	30510	6,976	3.1%
	Cystatin C (mg/L)	0.91	0.18	0.38	0.81	0.89	0.98	7.01	30720	13,177	5.8%
	Gamma-glutamyl transferase (U/L)	37.53	41.69	5.0	18.6	26.4	41.2	1167.2	30730	13,274	5.8%
	Glucose (mmol/L)	5.13	1.24	1.1	4.61	4.94	5.32	34.48	30740	32,095	14.1%
	Glycated haemoglobin (HbA1c, mmol/mol)	36.14	6.78	15.0	32.8	35.2	37.9	185	30750	14,066	6.2%
	LDL direct (mmol/L)	3.56	0.88	0.28	2.95	3.53	4.13	9.74	30780	13,564	5.9%
	Platelet count (10^9 cells/Litre)	253.24	60.1	0.3	213.4	248.1	287.2	1402	30080	9,898	4.3%
	Red blood cell count (10^12 cells/Litre)	4.51	0.42	0.01	4.23	4.5	4.79	7.79	30010	9,898	4.3%
	Serum creatinine (umol/L)	72.22	16.99	10.8	61.3	70.3	80.8	500	30700	13,259	5.8%
	Serum phosphate (mmol/L)	1.16	0.16	0.38	1.05	1.16	1.27	4.7	30810	32,174	14.1%
	Total bilirubin (umol/L)	9.11	4.4	1.43	6.41	8.06	10.4	100	30840	14,087	6.2%
	Total protein (g/L)	72.49	4.1	45.99	69.73	72.29	75.02	115.53	30860	32,058	14.0%
	Triglycerides (mmol/L)	1.76	1.03	0.24	1.05	1.49	2.16	11.28	30870	13,324	5.8%
	Urate (umol/L)	308.78	80.5	89.1	250	302.4	360.4	800	30880	13,436	5.9%

Supplementary Table 6: Missing value imputation details

Feature	N missing	% missing
**Non-biochemistry**		
Townsend deprivation index[1]	335	0.15%
Ethnicity	882	0.39%
Pulse rate (bpm)	233	0.10%
Systolic blood pressure (SBP)	235	0.10%
Diastolic blood pressure (DBP)	233	0.10%
Smoking	303	0.13%
Alcohol intake frequency	406	0.18%
Self-reported health	1,248	0.55%
Physical activity	1,498	0.66%
Home occupancy (lives alone)	1,668	0.73%
Post-secondary education	1,991	0.87%
SBP standard deviation	7,517	3.29%
**Biochemistry**		
Creatinine (enzymatic) in urine	6,976	3.06%
Platelet count	9,898	4.34%
Red blood cell (erythrocyte) count	9,898	4.34%
Alkaline phosphatase	13,139	5.76%
Total cholesterol	13,153	5.76%
Cystatin C	13,177	5.77%
Alanine aminotransferase	13,233	5.80%
Serum creatinine	13,259	5.81%
Gamma-glutamyl transferase	13,274	5.82%
Triglycerides	13,324	5.84%
Urate	13,436	5.89%
LDL direct	13,564	5.94%
C-reactive protein	13,606	5.96%
Aspartate aminotransferase	13,958	6.12%
HbA1cGlycated haemoglobin (HbA1c)	14,066	6.16%
Total bilirubin	14,087	6.17%
Albumin	31,839	13.95%
HDL cholesterol	31,961	14.00%
Total protein	32,058	14.05%
Glucose	32,095	14.06%
Serum phosphate	32,174	14.10%

Handling notes: 1. Townsend deprivation index missing values replaced with mean deprivation. 2. Ethnicity missing values were replaced with the overwhelming majority category "White". All other missingness was imputed together using multiple imputation with chained equations (MICE).

Supplementary Table 7: STROBE Checklist

STROBE Statement—Checklist of items that should be included in reports of ***cohort studies***

	Item No	Recommendation	Page
**Title and abstract**	1	(*a*) Indicate the study's design with a commonly used term in the title or the abstract	**2**
		(*b*) Provide in the abstract an informative and balanced summary of what was done and what was found	**2**
**Introduction**			
Background/rationale	2	Explain the scientific background and rationale for the investigation being reported	**4-5**
Objectives	3	State specific objectives, including any prespecified hypotheses	**5**
**Methods**			
Study design	4	Present key elements of study design early in the paper	**5-6**
Setting	5	Describe the setting, locations, and relevant dates, including periods of recruitment, exposure, follow-up, and data collection	**5**
Participants	6	(*a*) Give the eligibility criteria, and the sources and methods of selection of participants. Describe methods of follow-up	**5**
		(*b*) For matched studies, give matching criteria and number of exposed and unexposed	**N/A**
Variables	7	Clearly define all outcomes, exposures, predictors, potential confounders, and effect modifiers. Give diagnostic criteria, if applicable	**6-8**
Data sources/ measurement	8*	For each variable of interest, give sources of data and details of methods of assessment (measurement). Describe comparability of assessment methods if there is more than one group	***8, Supp tables 4 and 5***
Bias	9	Describe any efforts to address potential sources of bias	**Supplementary Methods SM1**
Study size	10	Explain how the study size was arrived at	**Supplementary Methods SM2, Supplementary Figure 1A**
Quantitative variables	11	Explain how quantitative variables were handled in the analyses. If applicable, describe which groupings were chosen and why	***8, Supp tables 4 and 5***
Statistical methods	12	(*a*) Describe all statistical methods, including those used to control for confounding	**8-9**
		(*b*) Describe any methods used to examine subgroups and interactions	**N/A**
		(*c*) Explain how missing data were addressed	**8, Supplementary Methods SM3: Supplementary Table 6**
		(*d*) If applicable, explain how loss to follow-up was addressed	**Supplementary Methods SM1, SM2, Supplementary Figure 1A**
		(*e*) Describe any sensitivity analyses	**9, Supp Fig 3 and Supp Table 11**
**Results**			
Participants	13*	(a) Report numbers of individuals at each stage of study—eg numbers potentially eligible, examined for eligibility, confirmed eligible, included in the study, completing follow-up, and analysed	**10, Supp Figure 1**
		(b) Give reasons for non-participation at each stage	
		(c) Consider use of a flow diagram	
Descriptive data	14*	(a) Give characteristics of study participants (eg demographic, clinical, social) and information on exposures and potential confounders	**Supp Tables 4 and 5**
		(b) Indicate number of participants with missing data for each variable of interest	

		(c) Summarise follow-up time (eg, average and total amount)	**Table 1**
Outcome data	15*	Report numbers of outcome events or summary measures over time	**Table 1**
Main results	16	(*a*) Give unadjusted estimates and, if applicable, confounder-adjusted estimates and their precision (eg, 95% confidence interval). Make clear which confounders were adjusted for and why they were included	**11, Supp Table 8**
		(*b*) Report category boundaries when continuous variables were categorized	**8**
		(*c*) If relevant, consider translating estimates of relative risk into absolute risk for a meaningful time period	**N/A although implicit in Supp Figure 3**
Other analyses	17	Report other analyses done—eg analyses of subgroups and interactions, and sensitivity analyses	**N/A**
**Discussion**			
Key results	18	Summarise key results with reference to study objectives	**13**
Limitations	19	Discuss limitations of the study, taking into account sources of potential bias or imprecision. Discuss both direction and magnitude of any potential bias	**13-14**
Interpretation	20	Give a cautious overall interpretation of results considering objectives, limitations, multiplicity of analyses, results from similar studies, and other relevant evidence	**13-14**
Generalisability	21	Discuss the generalisability (external validity) of the study results	**14**
**Other information**			
Funding	22	Give the source of funding and the role of the funders for the present study and, if applicable, for the original study on which the present article is based	**22**

*Give information separately for exposed and unexposed groups.

Note: An Explanation and Elaboration article discusses each checklist item and gives methodological background and published examples of transparent reporting. The STROBE checklist is best used in conjunction with this article (freely available on the Web sites of PLoS Medicine at http://www.plosmedicine.org/, Annals of Internal Medicine at http://www.annals.org/, and Epidemiology at http://www.epidem.com/). Information on the STROBE Initiative is available at http://www.strobe-statement.org.

Supplementary Figure 2: Stability retention threshold evaluations

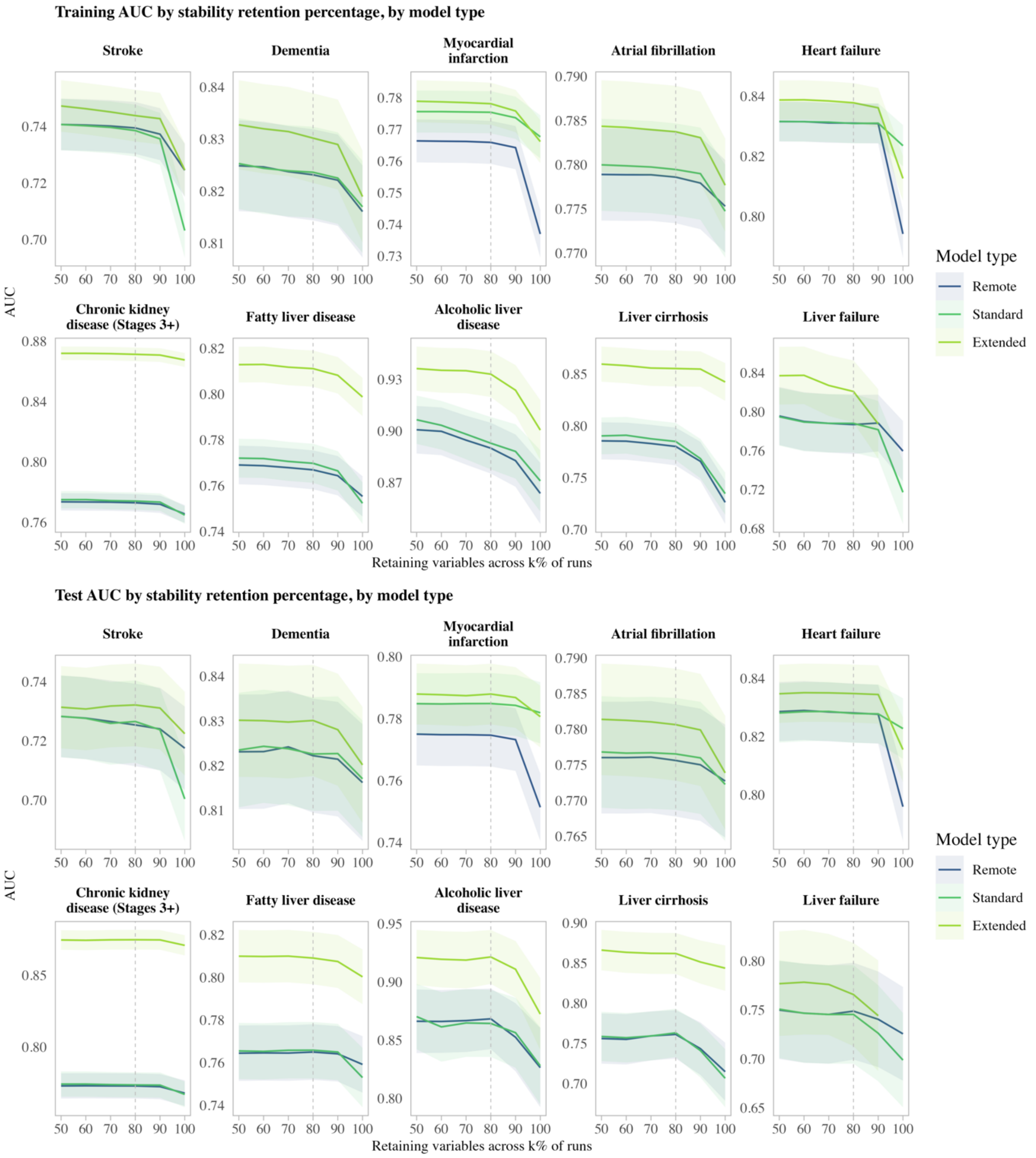

Supplementary Figure 2 footnote: Plots show the Training set and Test set AUC for models by levels of predictor retention from stability selection, for example, x=50 means that we retain predictors that were selected in at least 50% of the sample fits. The vertical dotted line shows the retention cutoff at 80/100

Supplementary Table 8: Details of pairwise Cox analysis

Category	Condition	Stroke	Dementia	Myocardial infarction	Atrial fibrillation	Heart failure	CKD (3+)	Fatty liver disease	Alcoholic liver disease	Liver cirrhosis	Liver failure
Risk	Hypertension	1.46*	1.32*	1.53*	1.56*	1.97*	1.80*	1.41*	2.41*	1.58*	1.61*
factors		[1.36, 1.57]	[1.21, 1.44]	[1.45, 1.63]	[1.49, 1.63]	[1.84, 2.11]	[1.71, 1.89]	[1.30, 1.52]	[1.98, 2.93]	[1.36, 1.84]	[1.25, 2.07]
		9.42x10-25	6.02x10-10	4.99x10-47	1.80x10-82	1.45x10-86	3.04x10-^{121}	3.89x10-18	8.64x10-19	2.59x10-9	2.44x10-4
	High cholesterol	1.03	1.19*	1.20*	1.09*	1.31*	1.23*	1.28*	0.83	0.93	1.02
		[0.95, 1.11]	[1.08, 1.30]	[1.13, 1.28]	[1.03, 1.14]	[1.23, 1.40]	[1.16, 1.29]	[1.18, 1.39]	[0.67, 1.03]	[0.79, 1.10]	[0.78, 1.34]
		0.5058	2.45x10-4	8.15x10-9	7.93x10-4	6.08x10-16	5.87x10-15	8.99x10-9	0.0962	0.3988	0.8741
	Diabetes	1.49*	1.75*	1.38*	1.13*	1.60*	1.91*	1.79*	2.26*	3.04*	2.57*
		[1.34, 1.66]	[1.56, 1.97]	[1.27, 1.50]	[1.05, 1.20]	[1.48, 1.73]	[1.79, 2.03]	[1.62, 1.98]	[1.75, 2.91]	[2.54, 3.64]	[1.89, 3.49]
		2.05x10-13	8.07x10-21	5.83x10-14	7.17x10-4	5.78x10-31	6.85x10-90	3.56x10-30	2.80x10-10	9.59x10-34	1.43x10-9
	COPD	1.19*	1.43*	1.54*	1.48*	1.94*	1.33*	1.26*	1.69*	1.26	1.97*
		[1.02, 1.39]	[1.21, 1.68]	[1.37, 1.73]	[1.35, 1.62]	[1.75, 2.16]	[1.20, 1.46]	[1.06, 1.49]	[1.21, 2.37]	[0.94, 1.69]	[1.29, 3.00]
		0.0262	1.85x10-5	3.73x10-13	1.36x10-17	5.34x10-35	3.18x10-8	0.0083	0.0021	0.1261	0.0016
	Any cancer	1.24*	1.08	1.10*	1.20*	1.30*	1.23*	1.19*	1.24	1.39*	1.84*
		[1.13, 1.36]	[0.97, 1.21]	[1.02, 1.20]	[1.13, 1.28]	[1.19, 1.41]	[1.16, 1.31]	[1.07, 1.32]	[0.94, 1.64]	[1.15, 1.68]	[1.37, 2.46]
		5.86x10-6	0.1683	0.0178	1.19x10-9	3.88x10-10	3.50x10-11	0.0017	0.1218	7.11x10-4	4.82x10-5
Arterial	Atherosclerosis /	1.52*	1.21	1.44*	1.46*	1.94*	1.52*	0.93	1.39	1.61*	2.05*
	peripheral artery disease	[1.31, 1.77]	[1.01, 1.45]	[1.26, 1.65]	[1.33, 1.61]	[1.74, 2.17]	[1.37, 1.68]	[0.76, 1.14]	[0.93, 2.06]	[1.21, 2.15]	[1.33, 3.17]
		4.47x10-8	0.0438	8.76x10-8	6.37x10-15	7.92x10-32	2.28x10-16	0.4923	0.104	0.0011	0.0011
	Venous thromboembolism	1.39*	1.32*	1.28*	1.47*	1.60*	1.22*	1.17*	1.77*	1.35*	1.69*
	(DVT/PE)	[1.23, 1.58]	[1.14, 1.54]	[1.14, 1.43]	[1.36, 1.58]	[1.45, 1.77]	[1.12, 1.33]	[1.02, 1.34]	[1.29, 2.43]	[1.05, 1.73]	[1.14, 2.50]
		2.87x10-7	2.17x10-4	1.32x10-5	1.51x10-22	4.69x10-20	3.05x10-6	0.027	4.35x10-4	0.0193	0.0087
	Erectile	1.11	1.20*	1.14*	1.14*	1.1	1.14*	1.12	1.38*	1.09	1.35
	dysfunction	[0.98, 1.25]	[1.05, 1.38]	[1.04, 1.24]	[1.06, 1.22]	[1.00, 1.21]	[1.05, 1.24]	[0.98, 1.28]	[1.06, 1.79]	[0.86, 1.38]	[0.95, 1.91]
		0.0878	0.0077	0.0051	3.85x10-4	0.0468	0.0017	0.1024	0.0173	0.4681	0.0972
Brain	Stroke		1.94*	1.1	1.45*	1.75*	1.30*	1.07	1.12	1.39	1.48
			[1.66, 2.27]	[0.96, 1.27]	[1.31, 1.60]	[1.56, 1.96]	[1.17, 1.44]	[0.88, 1.29]	[0.71, 1.74]	[1.02, 1.90]	[0.90, 2.44]
			7.18x10-17	0.1656	1.36x10-13	3.16x10-22	1.14x10-6	0.4958	0.6311	0.0353	0.1233
	Transient ischaemic	2.39*	1.65*	1.12	1.38*	1.57*	1.33*	1.19	0.83	1.15	1.25
	attack (TIA)	[1.89, 3.02]	[1.35, 2.02]	[0.94, 1.33]	[1.22, 1.56]	[1.36, 1.82]	[1.16, 1.51]	[0.94, 1.52]	[0.41, 1.67]	[0.73, 1.79]	[0.61, 2.55]
		4.33x10-13	1.13x10-6	0.2094	3.05x10-7	1.64x10-9	2.43x10-5	0.1568	0.5937	0.5529	0.5362
	Cerebrovascular diseases	1.86*	1.92*	1.27*	1.58*	1.66*	1.22*	1.22	2.12*	1.43	2.29*
		[1.29, 2.69]	[1.53, 2.42]	[1.04, 1.55]	[1.37, 1.83]	[1.40, 1.97]	[1.03, 1.43]	[0.92, 1.60]	[1.28, 3.52]	[0.91, 2.27]	[1.24, 4.23]
		8.62x10-4	2.64x10-8	0.0204	3.02x10-10	5.26x10-9	0.019	0.1651	0.0036	0.1248	0.0079
	Alzheimer's disease	2.49*		1.6	1.73*	1.18	1.36	1.06	6.40*	1.58	2.73
	/ dementia	[1.54, 4.01]		[1.03, 2.49]	[1.24, 2.43]	[0.70, 2.00]	[0.92, 2.02]	[0.51, 2.23]	[3.02, 13.53]	[0.51, 4.90]	[0.68, 11.00]
		1.81x10-4		0.0355	0.0014	0.529	0.1221	0.8723	1.20x10-6	0.4319	0.1577

Entries are hazard ratios, 95% confidence intervals and p-values for incident outcomes (outcomes shown along the top) associated with the presence of existing risk factors, diagnoses and medication at baseline (shown down the left-hand side) in the whole cohort (n= 228,240), using Cox-proportional hazards regression. For example, pre-existing hypertension increases the 10-year risk of stroke by 46%. Models are adjusted by age, sex, post-secondary education, ethnicity, smoking, physical activity, alcohol intake frequency, body mass index, Townsend deprivation index, family history (of heart disease, stroke and dementia), any cancer diagnosis, hypertension, high cholesterol and diabetes. Hazard ratio significance was adjusted for multiple testing with a false discovery rate of 5%, where significant results are indicated with an asterisk (*). Each result is from a different model.

Supplementary Table 8: Details of pairwise Cox analysis (continued)

Category	Condition	Stroke	Dementia	Myocardial infarction	Atrial fibrillation	Heart failure	CKD (3+)	Fatty liver disease	Alcoholic liver disease	Liver cirrhosis	Liver failure
	Epilepsy	1.86*	2.66*	1.41*	1.46*	1.18	1.06	1.04	2.17*	1.87*	2.09*
		[1.51, 2.30]	[2.17, 3.25]	[1.18, 1.69]	[1.27, 1.68]	[0.96, 1.46]	[0.90, 1.26]	[0.81, 1.35]	[1.37, 3.44]	[1.27, 2.75]	[1.14, 3.82]
		4.63x10-9	4.36x10-21	1.67x10-4	1.83x10-7	0.1161	0.4814	0.7421	9.46x10-4	0.0014	0.0165
	Parkinson's disease	1.37	9.14*	1.74*	1.55*	2.00*	1.62*	1.6	1.08	1.48	1.59
		[0.88, 2.12]	[7.44, 11.23]	[1.27, 2.38]	[1.21, 2.00]	[1.47, 2.72]	[1.24, 2.10]	[1.01, 2.54]	[0.27, 4.32]	[0.61, 3.56]	[0.39, 6.37]
		0.1643	9.07x10-99	6.03x10-4	6.33x10-4	1.00x10-5	3.53x10-4	0.0469	0.9179	0.385	0.5163
	Migraines	1.15*	1.08	0.99	1.09	0.94	1.12*	1.22*	0.71	0.96	1.38
		[1.02, 1.30]	[0.93, 1.25]	[0.89, 1.10]	[1.00, 1.17]	[0.84, 1.06]	[1.03, 1.21]	[1.09, 1.36]	[0.46, 1.09]	[0.74, 1.25]	[0.94, 2.02]
		0.019	0.2935	0.9031	0.0382	0.3011	0.0049	5.70x10-4	0.1206	0.774	0.0971
	Sleep disorder	1.08*	1.00	1.13*	1.10*	1.17*	1.08*	1.30*	1.64*	1.31*	1.38*
	(insomnia, sleep apnoea)	[1.01, 1.15]	[0.92, 1.08]	[1.07, 1.19]	[1.05, 1.14]	[1.10, 1.24]	[1.03, 1.13]	[1.21, 1.39]	[1.39, 1.95]	[1.15, 1.49]	[1.11, 1.72]
		0.0256	0.9907	7.73x10-6	9.04x10-6	9.43x10-8	8.12x10-4	7.65x10-14	9.92x10-9	5.66x10-5	0.0043
	Traumatic brain injury	1.32*	2.00*	1.19	1.11	1.25*	1.01	1.12	2.02*	1.1	1.45
		[1.08, 1.61]	[1.63, 2.44]	[1.01, 1.39]	[0.97, 1.27]	[1.05, 1.48]	[0.87, 1.18]	[0.90, 1.40]	[1.41, 2.90]	[0.72, 1.66]	[0.80, 2.66]
		0.0062	1.45x10-11	0.0353	0.1173	0.0131	0.8897	0.3017	1.24x10-4	0.6645	0.2246
Cardiac	Ischaemic heart disease	1.55*	1.38*	2.65*	1.52*	2.56*	1.30*	1.02	1.27	1.55*	1.68*
		[1.40, 1.71]	[1.23, 1.54]	[2.45, 2.87]	[1.43, 1.62]	[2.38, 2.76]	[1.22, 1.39]	[0.91, 1.15]	[0.97, 1.66]	[1.27, 1.88]	[1.22, 2.31]
		9.30x10-18	2.56x10-8	2.30x10^{-127}	9.30x10-42	2.11x10^{-141}	8.42x10-16	0.6909	0.0868	1.17x10-5	0.0016
	Myocardial infarction	1.62*	1.28*		1.46*	2.92*	1.37*	0.89	1.1	1.42*	1.73*
		[1.43, 1.83]	[1.11, 1.48]		[1.35, 1.58]	[2.69, 3.17]	[1.27, 1.49]	[0.76, 1.04]	[0.78, 1.56]	[1.11, 1.81]	[1.18, 2.54]
		1.80x10-14	7.76x10-4		2.86x10-22	3.92x10-^{143}	1.75x10-14	0.1482	0.5893	0.0055	0.005
	Cardiac arrhythmia	1.83*	1.34*	1.34*	2.47*	2.99*	1.43*	1.03	1.21	1.41*	1.66*
		[1.64, 2.04]	[1.17, 1.54]	[1.21, 1.49]	[2.28, 2.68]	[2.77, 3.23]	[1.32, 1.54]	[0.89, 1.20]	[0.86, 1.71]	[1.11, 1.80]	[1.14, 2.43]
		1.21x10-27	2.17x10-5	1.95x10-8	7.32x10^{-108}	2.91x10^{-168}	9.49x10-20	0.673	0.275	0.0053	0.0083
	Atrial fibrillation	2.52*	1.42*	1.31*		3.57*	1.52*	0.95	1.37	1.83*	1.81*
		[2.22, 2.85]	[1.19, 1.68]	[1.14, 1.49]		[3.25, 3.91]	[1.38, 1.68]	[0.77, 1.18]	[0.90, 2.09]	[1.38, 2.44]	[1.13, 2.91]
		3.77x10-47	6.90x10-5	1.02x10-4		9.92x10^{-163}	1.07x10-16	0.6424	0.1463	2.70x10-5	0.0135
	Non-ischaemic	1.91*	1.31	1.24	3.03*	5.50*	2.56*	0.93	1.72	1.27	2.98*
	cardiomyopathies	[1.35, 2.72]	[0.80, 2.15]	[0.86, 1.79]	[2.46, 3.74]	[4.41, 6.84]	[2.09, 3.14]	[0.56, 1.55]	[0.71, 4.15]	[0.57, 2.84]	[1.23, 7.25]
		2.67x10-4	0.2769	0.2478	5.52x10-25	2.44x10-52	1.87x10-19	0.7803	0.2313	0.5602	0.0159
	Valvular heart disease	1.96*	1.22	1.59*	2.94*	3.60*	1.56*	1.07	0.96	1.59*	2.33*
		[1.66, 2.31]	[0.97, 1.53]	[1.36, 1.85]	[2.66, 3.24]	[3.22, 4.04]	[1.39, 1.76]	[0.84, 1.36]	[0.51, 1.79]	[1.10, 2.31]	[1.38, 3.93]
		3.12x10-15	0.0967	3.56x10-9	3.60x10^{-103}	4.85x10^{-109}	2.10x10-13	0.6039	0.8911	0.0146	0.0015
	Heart failure	2.30*	1.42*	1.80*	2.49*		2.10*	1.14	1.13	1.84*	3.02*
		[1.90, 2.77]	[1.10, 1.82]	[1.44, 2.26]	[2.19, 2.82]		[1.85, 2.38]	[0.87, 1.48]	[0.60, 2.13]	[1.26, 2.69]	[1.80, 5.08]
		7.86x10-18	0.0068	3.27x10-7	2.34x10-44		2.46x10-30	0.3486	0.7067	0.0016	3.05x10-5
Kidney	Hypertensive renal disease	3.10*	1.36	2.56*	2.08*	2.56*	4.30*	0.88		2.18*	8.00*
		[2.16, 4.45]	[0.77, 2.41]	[1.90, 3.45]	[1.58, 2.72]	[1.90, 3.43]	[3.06, 6.03]	[0.47, 1.64]		[1.08, 4.40]	[4.20, 15.27]
		9.27x10-10	0.2868	6.56x10-10	1.31x10-7	4.53x10-10	3.13x10-17	0.6812		0.029	2.76x10-10

Supplementary Table 8: Details of pairwise Cox analysis (continued)

Category	Condition	Stroke	Dementia	Myocardial infarction	Atrial fibrillation	Heart failure	CKD (3+)	Fatty liver disease	Alcoholic liver disease	Liver cirrhosis	Liver failure
	Acute kidney injury	2.05*	1.71*	1.92*	1.88*	2.87*	3.65*	0.84	1.31	3.18*	5.60*
		[1.39, 3.01]	[1.08, 2.72]	[1.41, 2.63]	[1.47, 2.42]	[2.22, 3.70]	[2.82, 4.72]	[0.47, 1.53]	[0.42, 4.08]	[1.83, 5.52]	[2.75, 11.40]
		2.86x10-4	0.0235	4.08x10-5	7.30x10-7	7.16x10-16	4.91x10-23	0.5742	0.6449	3.91x10-5	1.98x10-6
	Chronic kidney disease	1.54*	1.14	1.21*	1.29*	1.51*		1.05	0.82	1.56*	3.57*
		[1.35, 1.77]	[0.97, 1.34]	[1.07, 1.37]	[1.18, 1.41]	[1.36, 1.67]		[0.89, 1.24]	[0.50, 1.37]	[1.20, 2.03]	[2.55, 5.00]
		2.46x10-10	0.1073	0.002	1.14x10-8	1.93x10-14		0.5481	0.4549	7.88x10-4	1.01x10-13
Liver	Fatty liver disease	1.29	1.12	1.39*	1.02	1.33*	1.31*			3.79*	2.46*
		[0.90, 1.84]	[0.72, 1.74]	[1.08, 1.80]	[0.81, 1.30]	[1.03, 1.72]	[1.07, 1.61]			[2.63, 5.45]	[1.16, 5.25]
		0.1596	0.6153	0.0116	0.8429	0.0304	0.0105			7.24x10-13	0.0194
	Alcoholic liver disease	1.6	4.49*	1.42	1.79*	1.63*	1.62*			16.15*	13.79*
		[0.99, 2.58]	[3.10, 6.49]	[0.98, 2.07]	[1.33, 2.41]	[1.13, 2.37]	[1.16, 2.26]			[11.67, 22.34]	[7.97, 23.86]
		0.0534	1.46x10-15	0.0634	1.25x10-4	0.0098	0.0044			3.05x10-63	6.24x10-21
	Liver cirrhosis	1.59	1.85*	1.2	1.34	1.88*	2.01*				28.85*
		[0.99, 2.56]	[1.09, 3.12]	[0.77, 1.86]	[0.95, 1.89]	[1.31, 2.71]	[1.52, 2.66]				[17.81, 46.73]
		0.0569	0.0224	0.4156	0.092	7.05x10-4	8.84x10-7				1.81x10-42
	Infective/viral hepatitis	0.93	0.91	0.96	1.27*	1.12	1	1.18	2.54*	3.22*	2.41*
		[0.69, 1.24]	[0.64, 1.31]	[0.76, 1.21]	[1.08, 1.49]	[0.88, 1.43]	[0.82, 1.21]	[0.89, 1.57]	[1.59, 4.06]	[2.30, 4.49]	[1.28, 4.52]
		0.6035	0.6129	0.7085	0.0035	0.3457	0.987	0.2447	1.03x10-4	6.51x10-12	0.0062
Systemic inflammation	Gout	1.15	0.97	1.09	1.28*	1.40*	1.44*	1.30*	2.04*	1.64*	1.73*
		[1.00, 1.33]	[0.81, 1.15]	[0.98, 1.22]	[1.18, 1.38]	[1.27, 1.55]	[1.31, 1.57]	[1.12, 1.50]	[1.55, 2.67]	[1.29, 2.07]	[1.18, 2.54]
		0.0476	0.7152	0.13	1.78x10-9	7.21x10-11	2.22x10-15	4.12x10-4	2.77x10-7	4.61x10-5	0.005
	Rheumatoid arthritis	1.46*	1.61*	1.66*	1.30*	1.77*	1.38*	1.39*	1.29	1.53*	1.79
		[1.20, 1.77]	[1.30, 1.99]	[1.42, 1.95]	[1.15, 1.48]	[1.52, 2.07]	[1.22, 1.57]	[1.13, 1.71]	[0.71, 2.34]	[1.05, 2.24]	[0.98, 3.27]
		1.90x10-4	1.50x10-5	1.68x10-10	5.97x10-5	1.47x10-13	7.47x10-7	0.0015	0.4096	0.0267	0.0595
	Systemic lupus erythematosus	2.85*	0.87	2.85*	1.62*	2.10*	2.40*	1.64		2.69	
		[1.77, 4.60]	[0.32, 2.31]	[1.86, 4.38]	[1.07, 2.44]	[1.29, 3.44]	[1.73, 3.31]	[0.91, 2.97]		[1.01, 7.20]	
		1.68x10-5	0.776	1.72x10-6	0.0211	0.003	1.17x10-7	0.1016		0.0488	
Mental illness	Anxiety disorders	1.12*	1.42*	1.08	1.07*	1.05	1.09*	1.42*	1.75*	1.36*	1.45*
		[1.03, 1.22]	[1.29, 1.57]	[1.00, 1.16]	[1.01, 1.13]	[0.97, 1.14]	[1.03, 1.16]	[1.31, 1.54]	[1.43, 2.15]	[1.16, 1.61]	[1.11, 1.91]
		0.0105	3.16x10-12	0.0422	0.0168	0.2104	0.0023	5.18x10-17	5.62x10-8	2.12x10-4	0.0074
	Serious mental illness	1.52*	3.17*	1.06	1.25*	1.44*	1.74*	1.01	1.73*	1.45	1.8
		[1.21, 1.92]	[2.58, 3.90]	[0.86, 1.31]	[1.06, 1.48]	[1.18, 1.77]	[1.49, 2.02]	[0.77, 1.31]	[1.04, 2.85]	[0.94, 2.25]	[0.93, 3.51]
		3.89x10-4	9.66x10-28	0.5902	0.0078	4.16x10-4	6.78x10-13	0.9697	0.0332	0.0912	0.0833
	Depression	1.17*	1.71*	1.19*	1.09*	1.20*	1.15*	1.49*	2.35*	1.50*	1.67*
		[1.09, 1.27]	[1.57, 1.86]	[1.12, 1.27]	[1.04, 1.15]	[1.12, 1.28]	[1.10, 1.21]	[1.39, 1.61]	[1.97, 2.81]	[1.29, 1.73]	[1.31, 2.12]
		4.46x10-5	1.58x10-34	6.68x10-8	5.63x10-4	1.44x10-7	5.03x10-8	1.01x10-26	4.55x10-21	4.72x10-8	2.78x10-5

Supplementary Table 9: Performance of existing risk indices in the whole sample

Outcome	Type	Risk score	Events / N	Prev	Thresh	AUC / C-statistic	Sens	Spec	Brier	Dxy	Missing
**Stroke**	Remote	QKidney 5	3,953 / 223,956	0.018	0.14	0.705 [0.697, 0.712]	0.682	0.628	0.018	0.406	4,284 (1.9%)
		CHARGE-AF			1.63	0.703 [0.695, 0.711]	0.682	0.626	0.019	0.406	
		Framingham (without blood)			15.35	0.701 [0.693, 0.709]	0.718	0.582	0.053	0.402	
	Standard	QStroke			4.58	0.726 [0.719, 0.734]	0.689	0.646	0.018	0.453	
		QRISK3			12.48	0.717 [0.709, 0.725]	0.697	0.627	0.034	0.434	
		QKidney 5			0.14	0.705 [0.697, 0.712]	0.682	0.628	0.018	0.406	
	Extended	QStroke			4.58	0.726 [0.719, 0.734]	0.689	0.646	0.018	0.453	
		QRISK3			12.48	0.717 [0.709, 0.725]	0.697	0.627	0.034	0.434	
		PCP-HF			2.53	0.705 [0.698, 0.713]	0.712	0.602	0.019	0.411	
**Dementia**	Remote	UKB-DRS	2,720 / 227,894	0.012	0.03	0.801 [0.794, 0.809]	0.788	0.682	0.014	0.603	346 (0.2%)
		CHARGE-AF			1.70	0.763 [0.755, 0.771]	0.776	0.637	0.014	0.525	
		QKidney 3			2.35	0.762 [0.755, 0.770]	0.780	0.626	0.015	0.525	
	Standard	UKB-DRS			0.03	0.801 [0.794, 0.809]	0.788	0.682	0.014	0.603	
		QStroke			4.70	0.781 [0.773, 0.788]	0.786	0.650	0.014	0.561	
		CHARGE-AF			1.70	0.763 [0.755, 0.771]	0.776	0.637	0.014	0.525	
	Extended	UKB-DRS			0.03	0.801 [0.794, 0.809]	0.788	0.682	0.014	0.603	
		QStroke			4.70	0.781 [0.773, 0.788]	0.786	0.650	0.014	0.561	
		CHARGE-AF			1.70	0.763 [0.755, 0.771]	0.776	0.637	0.014	0.525	
**Myocardial infarction**	Remote	Framingham (without blood)	5,953 / 221,576	0.027	17.23	0.730 [0.725, 0.736]	0.691	0.647	0.055	0.461	6,664 (3.0%)
		QKidney 5			0.11	0.707 [0.701, 0.714]	0.729	0.566	0.027	0.414	
		Cambridge Diabetes Score			27.03	0.690 [0.683, 0.696]	0.609	0.661	0.120	0.379	
	Standard	QRISK3			12.66	0.749 [0.743, 0.754]	0.727	0.645	0.039	0.497	
		Framingham (with blood)			14.68	0.737 [0.731, 0.743]	0.728	0.625	0.047	0.474	
		Framingham (without blood)			17.23	0.730 [0.725, 0.736]	0.691	0.647	0.055	0.461	
	Extended	QRISK3			12.66	0.749 [0.743, 0.754]	0.727	0.645	0.039	0.497	
		Framingham (with blood)			14.68	0.737 [0.731, 0.743]	0.728	0.625	0.047	0.474	
		Framingham (without blood)			17.23	0.730 [0.725, 0.736]	0.691	0.647	0.055	0.461	
**Atrial fibrillation**	Remote	CHARGE-AF	9,997 / 224,008	0.045	1.73	0.760 [0.755, 0.764]	0.729	0.663	0.041	0.519	4,232 (1.9%)
		QKidney 5			0.11	0.733 [0.728, 0.737]	0.782	0.574	0.044	0.463	
		Framingham (without blood)			18.20	0.728 [0.723, 0.732]	0.667	0.670	0.065	0.455	
	Standard	CHARGE-AF			1.73	0.760 [0.755, 0.764]	0.729	0.663	0.041	0.519	
		QStroke			4.06	0.746 [0.742, 0.751]	0.765	0.610	0.041	0.493	
		QKidney 5			0.11	0.733 [0.728, 0.737]	0.782	0.574	0.044	0.463	
	Extended	CHARGE-AF			1.73	0.760 [0.755, 0.764]	0.729	0.663	0.041	0.519	
		QStroke			4.06	0.746 [0.742, 0.751]	0.765	0.610	0.041	0.493	
		PCP-HF			2.56	0.740 [0.736, 0.745]	0.749	0.618	0.042	0.481	
**Heart failure**	Remote	QKidney 5	4,988 / 226,563	0.022	0.15	0.795 [0.789, 0.801]	0.777	0.659	0.022	0.589	1,677 (0.7%)
		QKidney 3			3.14	0.783 [0.777, 0.790]	0.697	0.720	0.021	0.567	
		CHARGE-AF			1.82	0.776 [0.769, 0.782]	0.743	0.670	0.024	0.551	
	Standard	QStroke			5.29	0.803 [0.797, 0.809]	0.751	0.710	0.022	0.606	
		QKidney 5			0.15	0.795 [0.789, 0.801]	0.777	0.659	0.022	0.589	
		QRISK3			13.45	0.785 [0.779, 0.791]	0.769	0.663	0.035	0.570	
	Extended	QStroke			5.29	0.803 [0.797, 0.809]	0.751	0.710	0.022	0.606	
		QKidney 5			0.15	0.795 [0.789, 0.801]	0.777	0.659	0.022	0.589	
		QRISK3			13.45	0.785 [0.779, 0.791]	0.769	0.663	0.035	0.570	

Table shows the three best-performing risk scores per outcome and per level of accessibility, by descending AUC.
Prev = Prevalence, Sens = Sensitivity, Spec = Specificity, Brier = Brier score, Dxy = Somers' Dxy

Supplementary Table 9: (Continued) Performance of existing risk indices in the whole sample

Outcome	Type	Risk score	Events / N	Preva-lence	Thresh	AUC / C-statistic	Sens	Spec	Brier	Dxy	Missing
**Chronic kidney disease**	Remote	QKidney 3	8,698 / 222,630	0.039	2.65	0.759 [0.754, 0.764]	0.700	0.684	0.036	0.518	5,610 (2.5%)
		QKidney 5			0.11	0.742 [0.737, 0.747]	0.772	0.591	0.038	0.484	
		UKB-DRS			0.02	0.731 [0.726, 0.736]	0.717	0.631	0.037	0.462	
	Standard	QKidney 3			2.65	0.759 [0.754, 0.764]	0.700	0.684	0.036	0.518	
		QKidney 5			0.11	0.742 [0.737, 0.747]	0.772	0.591	0.038	0.484	
		QStroke			4.41	0.741 [0.736, 0.746]	0.711	0.645	0.036	0.483	
	Extended	Kidney risk score			14.81	0.856 [0.852, 0.860]	0.785	0.777	0.051	0.711	
		QKidney 3			2.65	0.759 [0.754, 0.764]	0.700	0.684	0.036	0.518	
		QKidney 5			0.11	0.742 [0.737, 0.747]	0.772	0.591	0.038	0.484	
**Fatty liver disease**	Remote	Cambridge Diabetes Score	3,585 / 227,177	0.016	18.10	0.690 [0.682, 0.698]	0.755	0.536	0.124	0.380	1,063 (0.5%)
		LIBRA	3,585 / 227,177	0.016	2.65	0.680 [0.671, 0.688]	0.663	0.608	0.052	0.359	1,063 (0.5%)
		QKidney 5	3,585 / 227,177	0.016	0.21	0.626 [0.617, 0.636]	0.453	0.738	0.016	0.249	1,063 (0.5%)
	Standard	QDiabetes Model A	3,529 / 223,775	0.016	5.98	0.709 [0.701, 0.717]	0.733	0.576	0.028	0.419	4,465 (2.0%)
		QDiabetes Model C	3,585 / 227,177	0.016	3.92	0.692 [0.683, 0.701]	0.666	0.626	0.057	0.384	1,063 (0.5%)
		Cambridge Diabetes Score	3,585 / 227,177	0.016	18.10	0.690 [0.682, 0.698]	0.755	0.536	0.124	0.380	1,063 (0.5%)
	Extended	Dallas Steatosis Index	3,585 / 227,177	0.016	42.76	0.761 [0.754, 0.769]	0.742	0.656	0.175	0.522	1,063 (0.5%)
		Fatty liver index	3,585 / 227,177	0.016	59.72	0.759 [0.752, 0.766]	0.777	0.617	0.316	0.518	1,063 (0.5%)
		QDiabetes Model A	3,529 / 223,775	0.016	5.98	0.709 [0.701, 0.717]	0.733	0.576	0.028	0.419	4,465 (2.0%)
**Alcoholic liver disease**	Remote	Framingham (without blood)	561 / 227,806	0.002	15.80	0.711 [0.690, 0.731]	0.731	0.586	0.046	0.421	434 (0.2%)
		AUDIT-C (first question)	551 / 227,407	0.002	3.50	0.708 [0.684, 0.732]	0.601	0.804	0.451	0.416	833 (0.4%)
		LIBRA	561 / 227,806	0.002	2.10	0.700 [0.681, 0.719]	0.766	0.546	0.045	0.400	434 (0.2%)
	Standard	Framingham (without blood)	561 / 227,806	0.002	15.80	0.711 [0.690, 0.731]	0.731	0.586	0.046	0.421	434 (0.2%)
		AUDIT-C (first question)	551 / 227,407	0.002	3.50	0.708 [0.684, 0.732]	0.601	0.804	0.451	0.416	833 (0.4%)
		LIBRA	561 / 227,806	0.002	2.10	0.700 [0.681, 0.719]	0.766	0.546	0.045	0.400	434 (0.2%)
	Extended	AUDIT-C (three questions)	47 / 63,981	0.001	6.25	0.846 [0.783, 0.909]	0.830	0.777	0.187	0.692	164,259 (72%)
		APRI (AST / platelet ratio)	561 / 227,806	0.002	0.37	0.784 [0.761, 0.808]	0.620	0.857	0.005	0.568	434 (0.2%)
		Fatty liver index	561 / 227,806	0.002	70.08	0.731 [0.710, 0.752]	0.656	0.698	0.324	0.462	434 (0.2%)
**Liver cirrhosis**	Remote	LIBRA	951 / 227,742	0.004	2.55	0.693 [0.676, 0.710]	0.697	0.597	0.046	0.386	498 (0.2%)
		QKidney 5			0.15	0.693 [0.675, 0.710]	0.639	0.637	0.004	0.383	
		Cambridge Diabetes Score			38.45	0.686 [0.670, 0.703]	0.533	0.743	0.122	0.373	
	Standard	LIBRA			2.55	0.693 [0.676, 0.710]	0.697	0.597	0.046	0.386	
		QKidney 5			0.15	0.693 [0.675, 0.710]	0.639	0.637	0.004	0.383	
		Cambridge Diabetes Score			38.45	0.686 [0.670, 0.703]	0.533	0.743	0.122	0.373	
	Extended	APRI (AST / platelet ratio)			0.37	0.755 [0.736, 0.773]	0.569	0.856	0.006	0.509	
		Fatty liver index			69.24	0.730 [0.714, 0.747]	0.652	0.691	0.324	0.460	
		FIB-4			1.65	0.715 [0.696, 0.733]	0.533	0.798	0.050	0.429	
**Liver failure**	Remote	QKidney 5	340 / 227,985	0.001	0.21	0.701 [0.673, 0.729]	0.568	0.733	0.002	0.399	255 (0.1%)
		Framingham (without blood)			22.29	0.693 [0.665, 0.721]	0.553	0.738	0.046	0.385	
		Cambridge Diabetes Score			35.80	0.674 [0.645, 0.703]	0.541	0.721	0.122	0.348	
	Standard	QRISK3			10.43	0.701 [0.674, 0.728]	0.762	0.535	0.025	0.403	
		QKidney 5			0.21	0.701 [0.673, 0.729]	0.568	0.733	0.002	0.399	
		Framingham (without blood)			22.29	0.693 [0.665, 0.721]	0.553	0.738	0.046	0.385	
	Extended	QRISK3			10.43	0.701 [0.674, 0.728]	0.762	0.535	0.025	0.403	
		QKidney 5			0.21	0.701 [0.673, 0.729]	0.568	0.733	0.002	0.399	
		NAFLD Fibrosis Score			-1.05	0.698 [0.666, 0.730]	0.494	0.821	0.439	0.396	

AUC= area under the curve, Prev = prevalenc, Thresh = the threshold at which the listed index showed the highest balanced accuracy in the development cohort. Sens= sensitivity at given threshold, Spec= specificity at given threshold, Brier = Brier score, the average squared difference between predicted probabilities and outcomes, values closer to zero indicate better performance. Dxy = Somer’s Dxy statistic, the rank correlation between predicted probabilities and outcomes, values closer to one indicate better performance.

Supplementary Table 10: Summary of fitted model performance by bootstrapping in the validation cohort

Outcome	Type	Model	AUC / C-statistic	Brier score	Somers' Dxy	False negatives	False positives	Sensitivity	Specificity
**Stroke**	Remote	New	0.725 [0.711, 0.739]	0.018 [0.017, 0.019]	0.451 [0.423, 0.479]	390 [350, 430]	23,357 [23,107, 23,607]	0.682 [0.655, 0.708]	0.646 [0.642, 0.650]
		QKidney 5	0.701 [0.687, 0.715]	0.018 [0.017, 0.019]	0.309 [0.255, 0.363]	415 [374, 456]	23,629 [23,386, 23,872]	0.661 [0.635, 0.688]	0.642 [0.638, 0.645]
		Difference	0.024 [0.016, 0.033]	-0.000 [-0.001, 0.000]	0.142 [0.091, 0.193]	-25 [-52, 2]	-272 [-474, -70]	0.020 [-0.002, 0.043]	0.004 [0.001, 0.007]
	Standard	New	0.727 [0.713, 0.740]	0.018 [0.017, 0.019]	0.453 [0.426, 0.481]	388 [349, 427]	23,259 [23,020, 23,498]	0.683 [0.657, 0.709]	0.647 [0.644, 0.651]
		QStroke	0.727 [0.714, 0.741]	0.018 [0.017, 0.019]	0.455 [0.428, 0.481]	381 [344, 418]	23,236 [22,998, 23,474]	0.689 [0.664, 0.714]	0.648 [0.644, 0.651]
		Difference	-0.001 [-0.007, 0.005]	-0.000 [-0.001, 0.000]	-0.001 [-0.013, 0.011]	7.000 [-16, 30]	23 [-132, 178]	-0.006 [-0.025, 0.013]	-0.000 [-0.003, 0.002]
	Extended	New	0.732 [0.718, 0.746]	0.018 [0.017, 0.019]	0.464 [0.437, 0.492]	348 [313, 383]	24,458 [24,217, 24,699]	0.716 [0.691, 0.741]	0.629 [0.626, 0.633]
		QStroke	0.727 [0.714, 0.741]	0.018 [0.017, 0.019]	0.455 [0.427, 0.482]	381 [344, 418]	23,236 [22,999, 23,473]	0.689 [0.663, 0.715]	0.648 [0.644, 0.651]
		Difference	0.005 [-0.001, 0.011]	-0.000 [-0.001, -0.000]	0.010 [-0.003, 0.022]	-33 [-56, -10]	1,222 [1,054, 1,390]	0.027 [0.008, 0.046]	-0.019 [-0.021, -0.016]
**Dementia**	Remote	New	0.822 [0.810, 0.835]	0.011 [0.011, 0.012]	0.644 [0.620, 0.669]	184 [158, 210]	19,116 [18,890, 19,342]	0.773 [0.744, 0.801]	0.717 [0.714, 0.720]
		UKB-DRS	0.807 [0.793, 0.820]	0.012 [0.011, 0.012]	0.612 [0.586, 0.638]	202 [175, 229]	19,081 [18,859, 19,303]	0.750 [0.721, 0.779]	0.718 [0.714, 0.721]
		Difference	0.016 [0.009, 0.022]	-0.000 [-0.000, -0.000]	0.032 [0.019, 0.045]	-18 [-34, -2]	35 [-101, 171]	0.022 [0.003, 0.042]	-0.001 [-0.003, 0.001]
	Standard	New	0.823 [0.810, 0.836]	0.011 [0.011, 0.012]	0.645 [0.619, 0.671]	210 [182, 238]	17,160 [16,947, 17,373]	0.740 [0.711, 0.770]	0.746 [0.743, 0.749]
		UKB-DRS	0.807 [0.793, 0.820]	0.012 [0.011, 0.012]	0.612 [0.585, 0.640]	202 [174, 230]	19,081 [18,860, 19,302]	0.750 [0.720, 0.781]	0.718 [0.714, 0.721]
		Difference	0.016 [0.009, 0.023]	-0.000 [-0.000, -0.000]	0.033 [0.020, 0.046]	8 [-10, 26]	-1,921 [-2,061, -1,781]	-0.010 [-0.032, 0.013]	0.028 [0.026, 0.031]
	Extended	New	0.830 [0.818, 0.842]	0.011 [0.011, 0.012]	0.660 [0.636, 0.685]	226 [198, 254]	15,670 [15,443, 15,897]	0.721 [0.691, 0.750]	0.768 [0.765, 0.771]
		UKB-DRS	0.807 [0.793, 0.820]	0.012 [0.011, 0.012]	0.612 [0.586, 0.639]	202 [175, 229]	19,081 [18,849, 19,313]	0.750 [0.721, 0.780]	0.718 [0.714, 0.721]
		Difference	0.023 [0.015, 0.032]	-0.000 [-0.000, -0.000]	0.048 [0.032, 0.064]	24 [3, 45]	-3,411 [-3,569, -3,253]	-0.030 [-0.055, -0.004]	0.050 [0.048, 0.053]
**Myocardial infarction**	Remote	New	0.775 [0.765, 0.785]	0.026 [0.025, 0.027]	0.549 [0.529, 0.570]	491 [447, 535]	20,873 [20,640, 21,106]	0.732 [0.711, 0.752]	0.677 [0.674, 0.681]
		FRS (BMI)	0.736 [0.726, 0.747]	0.027 [0.026, 0.028]	0.472 [0.451, 0.493]	556 [508, 604]	22,769 [22,525, 23,013]	0.696 [0.675, 0.718]	0.648 [0.644, 0.652]
		Difference	0.039 [0.032, 0.045]	-0.001 [-0.002, -0.001]	0.077 [0.064, 0.090]	-65 [-99, -31]	-1,896 [-2,082, -1,710]	0.035 [0.017, 0.054]	0.029 [0.026, 0.032]
	Standard	New	0.785 [0.775, 0.795]	0.026 [0.025, 0.027]	0.570 [0.551, 0.589]	539 [496, 582]	18,902 [18,676, 19,128]	0.706 [0.686, 0.726]	0.708 [0.704, 0.711]
		QRISK3	0.757 [0.747, 0.767]	0.026 [0.025, 0.028]	0.514 [0.494, 0.535]	480 [438, 522]	23,306 [23,063, 23,549]	0.738 [0.718, 0.758]	0.640 [0.636, 0.643]
		Difference	0.028 [0.022, 0.034]	-0.001 [-0.001, -0.000]	0.055 [0.044, 0.067]	59 [27, 91]	-4,404 [-4,586, -4,222]	-0.032 [-0.050, -0.014]	0.068 [0.065, 0.071]
	Extended	New	0.788 [0.778, 0.798]	0.026 [0.025, 0.027]	0.576 [0.556, 0.596]	424 [385, 463]	22,106 [21,867, 22,345]	0.768 [0.749, 0.788]	0.658 [0.655, 0.662]
		QRISK3	0.757 [0.747, 0.768]	0.026 [0.025, 0.027]	0.514 [0.494, 0.535]	480 [439, 521]	23,306 [23,065, 23,547]	0.738 [0.718, 0.758]	0.640 [0.636, 0.643]
		Difference	0.031 [0.025, 0.037]	-0.001 [-0.001, -0.000]	0.062 [0.049, 0.074]	-56 [-88, -24]	-1,200 [-1,380, -1,020]	0.031 [0.013, 0.048]	0.019 [0.016, 0.021]
**Atrial fibrillation**	Remote	New	0.776 [0.768, 0.784]	0.041 [0.039, 0.042]	0.551 [0.535, 0.567]	793 [738, 848]	20,768 [20,528, 21,008]	0.737 [0.722, 0.753]	0.676 [0.673, 0.680]
		CHARGE-AF	0.759 [0.751, 0.767]	0.043 [0.041, 0.044]	0.518 [0.502, 0.534]	826 [770, 882]	21,457 [21,221, 21,693]	0.726 [0.710, 0.743]	0.666 [0.662, 0.669]
		Difference	0.017 [0.013, 0.020]	-0.002 [-0.003, -0.002]	0.034 [0.026, 0.041]	-33 [-67, 1]	-689 [-841, -537]	0.011 [-0.000, 0.022]	0.011 [0.008, 0.013]
	Standard	New	0.777 [0.768, 0.785]	0.041 [0.039, 0.042]	0.553 [0.537, 0.569]	822 [764, 880]	20,150 [19,923, 20,377]	0.728 [0.712, 0.744]	0.686 [0.683, 0.690]
		CHARGE-AF	0.759 [0.751, 0.767]	0.043 [0.041, 0.044]	0.518 [0.501, 0.535]	826 [769, 883]	21,457 [21,224, 21,690]	0.726 [0.710, 0.743]	0.666 [0.662, 0.669]
		Difference	0.018 [0.014, 0.021]	-0.002 [-0.003, -0.002]	0.035 [0.028, 0.043]	-4 [-39, 31]	-1,307 [-1,454, -1,160]	0.001 [-0.010, 0.013]	0.020 [0.018, 0.023]
	Extended	New	0.781 [0.773, 0.788]	0.041 [0.039, 0.042]	0.561 [0.546, 0.577]	813 [757, 869]	19,280 [19,046, 19,514]	0.731 [0.715, 0.746]	0.700 [0.696, 0.703]
		CHARGE-AF	0.759 [0.751, 0.767]	0.043 [0.041, 0.044]	0.518 [0.502, 0.534]	826 [769, 883]	21,457 [21,219, 21,695]	0.726 [0.710, 0.743]	0.666 [0.662, 0.669]
		Difference	0.022 [0.018, 0.026]	-0.002 [-0.003, -0.002]	0.044 [0.036, 0.052]	-13 [-51, 25]	-2,177 [-2,339, -2,015]	0.004 [-0.008, 0.017]	0.034 [0.031, 0.036]
**Heart failure**	Remote	New	0.828 [0.818, 0.838]	0.020 [0.019, 0.021]	0.656 [0.637, 0.676]	356 [317, 395]	17,580 [17,372, 17,788]	0.761 [0.738, 0.783]	0.736 [0.733, 0.739]
		QKidney 5	0.798 [0.787, 0.809]	0.022 [0.021, 0.023]	0.580 [0.555, 0.604]	381 [344, 418]	20,130 [19,898, 20,362]	0.744 [0.722, 0.765]	0.697 [0.694, 0.701]
		Difference	0.030 [0.024, 0.036]	-0.002 [-0.002, -0.001]	0.077 [0.061, 0.093]	-25 [-53, 3]	-2,550 [-2,745, -2,355]	0.017 [-0.002, 0.036]	0.038 [0.035, 0.041]
	Standard	New	0.828 [0.818, 0.838]	0.020 [0.019, 0.021]	0.656 [0.636, 0.676]	438 [398, 478]	14,303 [14,084, 14,522]	0.706 [0.683, 0.729]	0.785 [0.782, 0.788]
		QStroke	0.806 [0.795, 0.817]	0.021 [0.020, 0.022]	0.612 [0.590, 0.634]	363 [325, 401]	19,291 [19,054, 19,528]	0.756 [0.734, 0.778]	0.710 [0.706, 0.713]
		Difference	0.022 [0.016, 0.028]	-0.001 [-0.001, -0.000]	0.044 [0.033, 0.055]	75 [45, 105]	-4,988 [-5,173, -4,803]	-0.050 [-0.070, -0.031]	0.075 [0.072, 0.078]
	Extended	New	0.835 [0.825, 0.845]	0.020 [0.019, 0.021]	0.670 [0.650, 0.689]	365 [327, 403]	16,557 [16,336, 16,778]	0.755 [0.733, 0.776]	0.751 [0.748, 0.754]
		QStroke	0.806 [0.795, 0.817]	0.021 [0.020, 0.022]	0.612 [0.591, 0.633]	363 [326, 400]	19,291 [19,060, 19,522]	0.756 [0.735, 0.777]	0.710 [0.706, 0.713]
		Difference	0.029 [0.023, 0.035]	-0.001 [-0.001, -0.001]	0.058 [0.046, 0.070]	2 [-27, 31]	-2,734 [-2,913, -2,555]	-0.001 [-0.021, 0.018]	0.041 [0.038, 0.044]
**Chronic kidney disease**	Remote	New	0.773 [0.764, 0.782]	0.035 [0.034, 0.037]	0.546 [0.528, 0.563]	667 [618, 716]	21,425 [21,194, 21,656]	0.742 [0.725, 0.758]	0.666 [0.663, 0.670]
		QKidney 3	0.760 [0.751, 0.769]	0.037 [0.036, 0.039]	0.520 [0.502, 0.538]	790 [736, 844]	20,214 [19,983, 20,445]	0.694 [0.677, 0.711]	0.685 [0.681, 0.689]
		Difference	0.013 [0.009, 0.016]	-0.002 [-0.003, -0.002]	0.026 [0.018, 0.033]	-123 [-157, -89]	1,211 [1,052, 1,370]	0.048 [0.035, 0.061]	-0.019 [-0.021, -0.016]
	Standard	New	0.774 [0.765, 0.783]	0.035 [0.034, 0.037]	0.547 [0.530, 0.565]	795 [743, 847]	18,227 [17,992, 18,462]	0.692 [0.675, 0.709]	0.716 [0.712, 0.720]
		QKidney 3	0.760 [0.751, 0.769]	0.037 [0.036, 0.039]	0.520 [0.502, 0.538]	790 [734, 846]	20,214 [19,979, 20,449]	0.694 [0.677, 0.712]	0.685 [0.681, 0.689]
		Difference	0.014 [0.010, 0.017]	-0.002 [-0.003, -0.002]	0.027 [0.020, 0.035]	5 [-29, 39]	-1,987 [-2,140, -1,834]	-0.002 [-0.015, 0.011]	0.031 [0.029, 0.033]
	Extended	New	0.875 [0.868, 0.881]	0.031 [0.030, 0.032]	0.749 [0.736, 0.763]	500 [454, 546]	13,864 [13,670, 14,058]	0.806 [0.790, 0.823]	0.784 [0.781, 0.787]
		KRS	0.858 [0.851, 0.866]	0.035 [0.034, 0.036]	0.717 [0.702, 0.732]	539 [493, 585]	14,138 [13,931, 14,345]	0.791 [0.775, 0.807]	0.780 [0.776, 0.783]
		Difference	0.016 [0.013, 0.020]	-0.004 [-0.004, -0.004]	0.033 [0.025, 0.040]	-39 [-70, -8]	-274 [-423, -125]	0.015 [0.003, 0.027]	0.004 [0.002, 0.007]

Supplementary Table 10: (Continued) Summary of fitted model performance by bootstrapping in the validation cohort

Outcome	Type	Model	AUC / C-statistic	Brier score	Somers' Dxy	False negatives	False positives	Sensitivity	Specificity
**Fatty liver disease**	Remote	New	0.765 [0.752, 0.778]	0.017 [0.016, 0.019]	0.530 [0.504, 0.557]	295 [262, 328]	21,282 [21,037, 21,527]	0.719 [0.692, 0.746]	0.683 [0.679, 0.686]
		Cambridge	0.684 [0.670, 0.699]	0.057 [0.056, 0.059]	0.369 [0.340, 0.398]	276 [245, 307]	31,021 [30,762, 31,280]	0.737 [0.712, 0.763]	0.538 [0.534, 0.541]
		Difference	0.081 [0.068, 0.094]	-0.040 [-0.041, -0.038]	0.161 [0.136, 0.187]	19 [-11, 49]	-9,739 [-9,975, -9,503]	-0.018 [-0.046, 0.010]	0.145 [0.142, 0.149]
	Standard	New	0.766 [0.753, 0.779]	0.017 [0.015, 0.018]	0.532 [0.506, 0.558]	315 [280, 350]	19,786 [19,555, 20,017]	0.695 [0.667, 0.723]	0.701 [0.697, 0.704]
		QDiabetes A	0.704 [0.690, 0.719]	0.016 [0.015, 0.017]	0.409 [0.379, 0.438]	309 [274, 344]	26,804 [26,556, 27,052]	0.701 [0.673, 0.728]	0.594 [0.591, 0.598]
		Difference	0.061 [0.050, 0.073]	0.001 [-0.000, 0.001]	0.123 [0.101, 0.145]	6 [-25, 37]	-7,018 [-7,240, -6,796]	-0.006 [-0.036, 0.024]	0.106 [0.103, 0.110]
	Extended	New	0.809 [0.797, 0.822]	0.015 [0.014, 0.016]	0.618 [0.593, 0.643]	278 [246, 310]	18,646 [18,414, 18,878]	0.735 [0.708, 0.763]	0.722 [0.719, 0.725]
		Dallas SI	0.761 [0.747, 0.775]	0.049 [0.048, 0.051]	0.522 [0.493, 0.550]	277 [247, 307]	22,891 [22,647, 23,135]	0.736 [0.710, 0.763]	0.659 [0.655, 0.662]
		Difference	0.048 [0.039, 0.058]	-0.035 [-0.036, -0.033]	0.096 [0.078, 0.115]	1 [-27, 29]	-4,245 [-4,464, -4,026]	-0.001 [-0.027, 0.025]	0.063 [0.060, 0.067]
**Alcoholic liver disease**	Remote	New	0.868 [0.842, 0.895]	0.002 [0.002, 0.003]	0.734 [0.680, 0.787]	41 [29, 53]	13,485 [13,279, 13,691]	0.742 [0.674, 0.810]	0.802 [0.799, 0.805]
		FRS (BMI)	0.703 [0.656, 0.750]	0.005 [0.002, 0.007]	0.407 [0.312, 0.501]	46 [29, 63]	28,597 [28,399, 28,795]	0.711 [0.633, 0.788]	0.581 [0.578, 0.583]
		Difference	0.165 [0.118, 0.212]	-0.002 [-0.005, 0.000]	0.327 [0.233, 0.422]	-5.000 [-19, 8]	-15,112 [-15,307, -14,917]	0.031 [-0.046, 0.109]	0.222 [0.219, 0.225]
	Standard	New	0.864 [0.835, 0.894]	0.002 [0.002, 0.003]	0.726 [0.666, 0.787]	23 [14, 32]	17,876 [17,663, 18,089]	0.855 [0.799, 0.912]	0.738 [0.735, 0.741]
		FRS (BMI)	0.703 [0.656, 0.751]	0.005 [0.002, 0.007]	0.407 [0.312, 0.501]	46 [30, 62]	28,597 [28,394, 28,800]	0.711 [0.632, 0.790]	0.581 [0.578, 0.584]
		Difference	0.161 [0.110, 0.212]	-0.002 [-0.005, 0.000]	0.320 [0.217, 0.423]	-23 [-37, -8.623]	-10,721 [-10,930, -10,512]	0.145 [0.064, 0.225]	0.157 [0.154, 0.160]
	Extended	New	0.922 [0.899, 0.944]	0.002 [0.002, 0.003]	0.844 [0.801, 0.888]	37 [25, 49]	5,578 [5,443, 5,713]	0.767 [0.703, 0.831]	0.918 [0.916, 0.920]
		APRI	0.780 [0.735, 0.824]	0.002 [0.002, 0.003]	0.558 [0.471, 0.646]	67 [51, 83]	9,568 [9,380, 9,756]	0.579 [0.502, 0.655]	0.860 [0.857, 0.862]
		Difference	0.142 [0.101, 0.183]	-0.000 [-0.000, 0.000]	0.286 [0.206, 0.367]	-30 [-44, -16]	-3,990 [-4,191, -3,789]	0.189 [0.109, 0.268]	0.059 [0.056, 0.061]
**Liver cirrhosis**	Remote	New	0.762 [0.730, 0.793]	0.004 [0.003, 0.004]	0.523 [0.461, 0.585]	83 [65, 101]	21,445 [21,203, 21,687]	0.688 [0.631, 0.745]	0.685 [0.681, 0.688]
		LIBRA	0.662 [0.628, 0.696]	0.008 [0.007, 0.008]	0.324 [0.256, 0.392]	107 [87, 127]	25,448 [25,189, 25,707]	0.598 [0.542, 0.654]	0.626 [0.622, 0.630]
		Difference	0.100 [0.070, 0.129]	-0.004 [-0.004, -0.003]	0.199 [0.141, 0.257]	-24 [-41, -7]	-4,003 [-4,255, -3,751]	0.090 [0.029, 0.151]	0.059 [0.055, 0.063]
	Standard	New	0.763 [0.734, 0.793]	0.004 [0.003, 0.004]	0.526 [0.467, 0.586]	102 [83, 121]	15,368 [15,149, 15,587]	0.617 [0.559, 0.674]	0.774 [0.771, 0.777]
		LIBRA	0.662 [0.626, 0.698]	0.008 [0.007, 0.008]	0.324 [0.252, 0.396]	107 [86, 128]	25,448 [25,197, 25,699]	0.598 [0.535, 0.660]	0.626 [0.622, 0.630]
		Difference	0.101 [0.070, 0.133]	-0.004 [-0.004, -0.003]	0.202 [0.140, 0.265]	-5 [-21, 11]	-10,080 [-10,332, -9,828]	0.019 [-0.042, 0.080]	0.148 [0.144, 0.152]
	Extended	New	0.862 [0.837, 0.888]	0.004 [0.003, 0.004]	0.723 [0.672, 0.774]	79 [62, 96]	8,973 [8,806, 9,140]	0.703 [0.647, 0.759]	0.868 [0.866, 0.871]
		APRI	0.743 [0.711, 0.774]	0.004 [0.002, 0.006]	0.483 [0.420, 0.545]	113 [94, 132]	9,661 [9,425, 9,897]	0.575 [0.517, 0.633]	0.858 [0.855, 0.861]
		Difference	0.120 [0.091, 0.148]	-0.000 [-0.002, 0.002]	0.241 [0.183, 0.298]	-34 [-50, -18]	-688 [-938, -438]	0.128 [0.067, 0.188]	0.010 [0.006, 0.014]
**Liver failure**	Remote	New	0.749 [0.698, 0.800]	0.002 [0.001, 0.002]	0.489 [0.386, 0.593]	42 [30, 54]	15,874 [15,657, 16,091]	0.596 [0.501, 0.691]	0.768 [0.764, 0.771]
		QKidney 5	0.669 [0.616, 0.722]	0.002 [0.001, 0.002]	0.137 [-0.013, 0.287]	52 [39, 65]	17,896 [17,670, 18,122]	0.500 [0.402, 0.598]	0.738 [0.735, 0.741]
		Difference	0.080 [0.025, 0.134]	0.000 [-0.000, 0.000]	0.353 [0.189, 0.516]	-10 [-22, 2]	-2,022 [-2,286, -1,758]	0.096 [-0.023, 0.215]	0.030 [0.026, 0.033]
	Standard	New	0.746 [0.695, 0.796]	0.002 [0.001, 0.002]	0.494 [0.392, 0.596]	53 [39, 67]	11,372 [11,181, 11,563]	0.490 [0.396, 0.584]	0.833 [0.831, 0.836]
		QRISK3	0.686 [0.632, 0.740]	0.002 [0.002, 0.002]	0.373 [0.224, 0.521]	31 [17, 45]	31,779 [31,556, 32,002]	0.702 [0.607, 0.797]	0.535 [0.531, 0.538]
		Difference	0.059 [0.008, 0.111]	-0.000 [-0.000, -0.000]	0.121 [-0.035, 0.277]	22 [10, 34]	-20,407 [-20,645, -20,169]	-0.212 [-0.327, -0.096]	0.299 [0.295, 0.302]
	Extended	New	0.766 [0.714, 0.818]	0.001 [0.001, 0.002]	0.526 [0.421, 0.631]	50 [36, 64]	9,534 [9,359, 9,709]	0.519 [0.425, 0.614]	0.860 [0.858, 0.863]
		QRISK3	0.686 [0.626, 0.746]	0.002 [0.002, 0.002]	0.373 [0.253, 0.492]	31 [16, 46]	31,779 [31,590, 31,968]	0.702 [0.611, 0.793]	0.535 [0.532, 0.537]
		Difference	0.080 [0.030, 0.129]	-0.000 [-0.001, -0.000]	0.153 [0.053, 0.253]	19 [10, 28]	-22,245 [-22,456, -22,034]	-0.183 [-0.266, -0.099]	0.326 [0.323, 0.329]

AUC= area under the curve, Thresh = the threshold at which the listed index showed the highest balanced accuracy in the development cohort. Sens= sensitivity at given threshold, Spec= specificity at given threshold, Brier = Brier score, the average squared difference between predicted probabilities and outcomes, values closer to zero indicate better performance. Dxy = Somer's Dxy statistic, the rank correlation between predicted probabilities and outcomes, values closer to one indicate better performance.

Supplementary Figure 3: Calibration plots

Remote models: Models that can be applied fully remotely

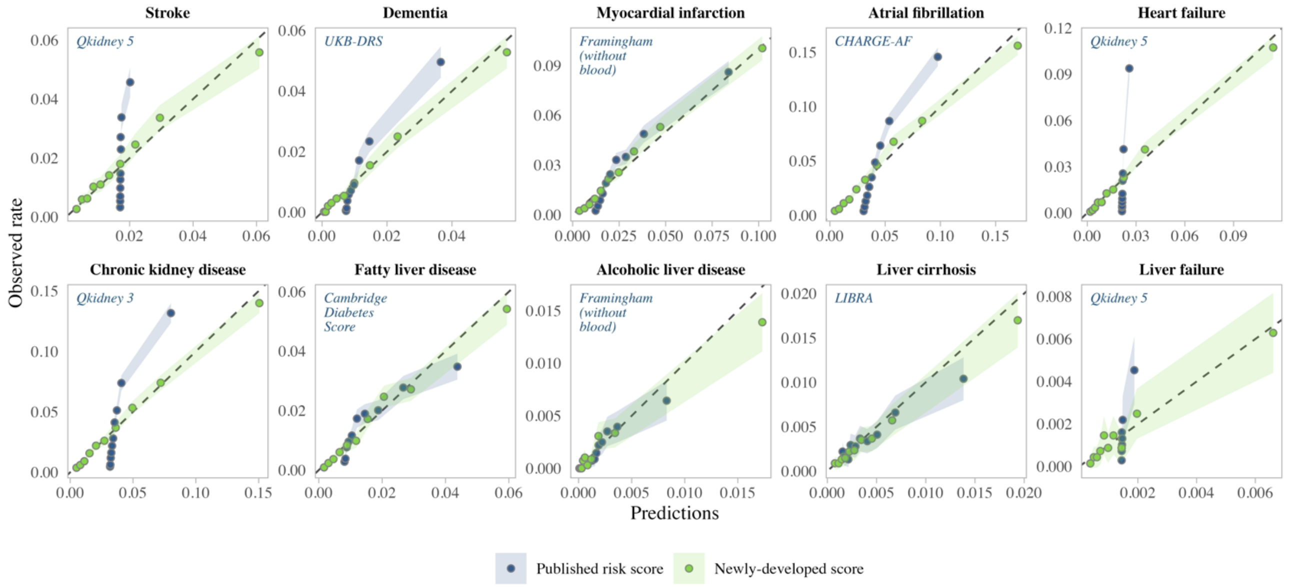

Standard models: Models limited to NHS Health Check / QRISK3 inputs

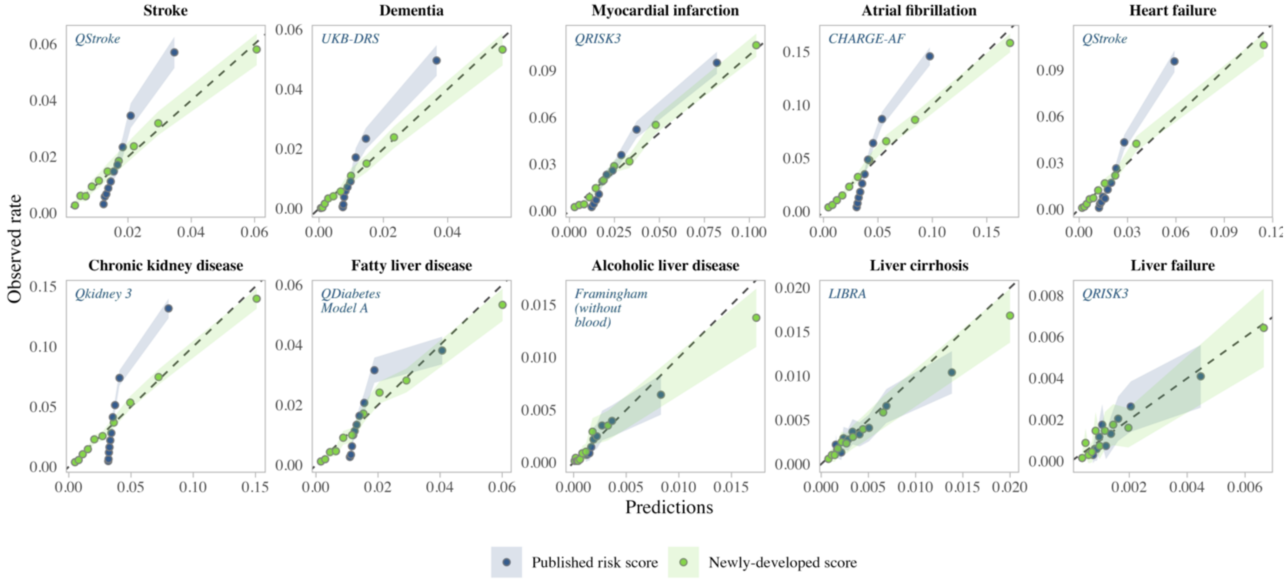

Extended models: Models including additional blood tests

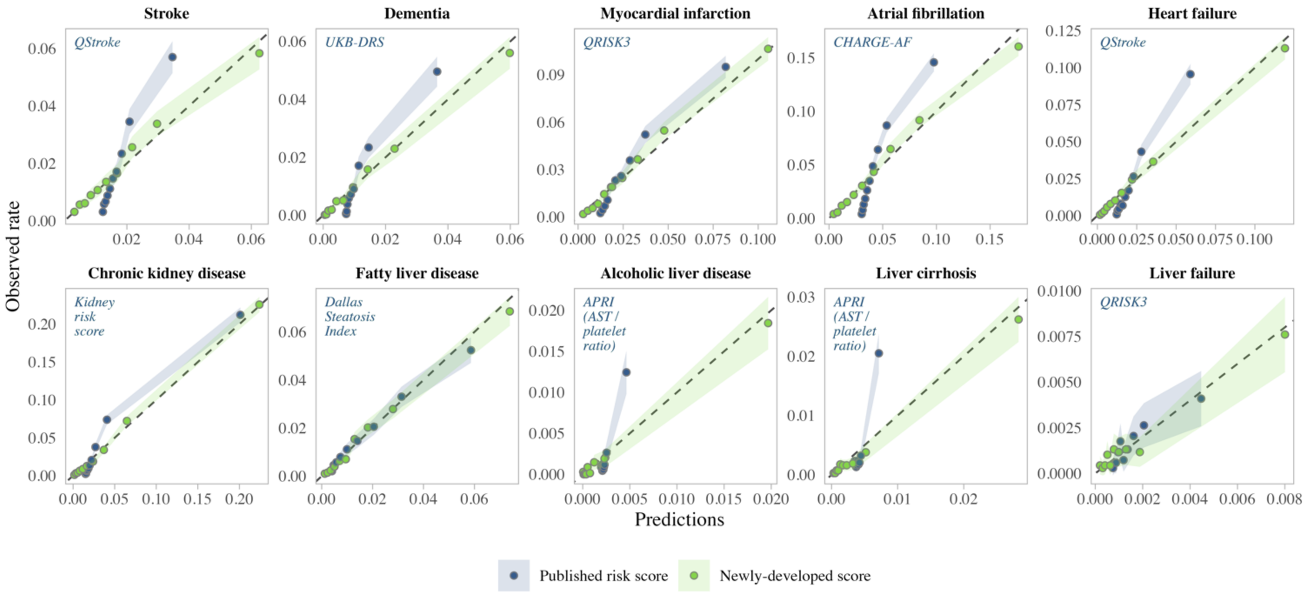

Supplementary Table 11: Reclassification indices

Comparing newly-developed risk models with best existing risk model of the same type

Outcome	Index	Remote models	Standard models	Extended models
**Stroke**	IDI	0.017* [0.015, 0.019]	0.011* [0.009, 0.013]	0.013* [0.010, 0.015]
	NRI events	0.337* [0.284, 0.390]	0.290* [0.236, 0.343]	0.311* [0.258, 0.364]
	NRI non-events	0.325* [0.318, 0.333]	0.319* [0.312, 0.326]	0.337* [0.330, 0.344]
	NRI overall	0.662* [0.609, 0.716]	0.609* [0.555, 0.663]	0.648* [0.594, 0.702]
**Dementia**	IDI	0.014* [0.009, 0.018]	0.014* [0.009, 0.018]	0.018* [0.013, 0.023]
	NRI events	0.473* [0.413, 0.534]	0.459* [0.397, 0.520]	0.434* [0.372, 0.496]
	NRI non-events	0.363* [0.356, 0.370]	0.367* [0.360, 0.374]	0.407* [0.400, 0.414]
	NRI overall	0.836* [0.775, 0.897]	0.826* [0.764, 0.887]	0.841* [0.778, 0.903]
**Myocardial infarction**	IDI	0.019* [0.016, 0.021]	0.014* [0.011, 0.017]	0.016* [0.013, 0.019]
	NRI events	0.253* [0.209, 0.297]	0.293* [0.249, 0.337]	0.290* [0.246, 0.334]
	NRI non-events	0.364* [0.357, 0.371]	0.339* [0.332, 0.347]	0.356* [0.349, 0.363]
	NRI overall	0.617* [0.572, 0.662]	0.633* [0.588, 0.677]	0.646* [0.602, 0.691]
**Atrial fibrillation**	IDI	0.038* [0.035, 0.040]	0.038* [0.036, 0.041]	0.043* [0.040, 0.046]
	NRI events	0.445* [0.413, 0.477]	0.446* [0.414, 0.478]	0.442* [0.410, 0.474]
	NRI non-events	0.344* [0.337, 0.351]	0.346* [0.339, 0.353]	0.369* [0.361, 0.376]
	NRI overall	0.789* [0.756, 0.822]	0.792* [0.759, 0.825]	0.810* [0.778, 0.843]
**Heart failure**	IDI	0.076* [0.069, 0.083]	0.043* [0.038, 0.049]	0.053* [0.046, 0.059]
	NRI events	0.464* [0.419, 0.509]	0.293* [0.244, 0.342]	0.337* [0.290, 0.385]
	NRI non-events	0.518* [0.511, 0.524]	0.554* [0.548, 0.560]	0.559* [0.553, 0.566]
	NRI overall	0.981* [0.936, 1.027]	0.847* [0.798, 0.896]	0.897* [0.848, 0.945]
**Chronic kidney disease (Stages 3, 4 and 5)**	IDI	0.031* [0.028, 0.034]	0.031* [0.028, 0.035]	0.032* [0.028, 0.037]
	NRI events	0.414* [0.379, 0.450]	0.398* [0.363, 0.434]	0.338* [0.301, 0.374]
	NRI non-events	0.337* [0.330, 0.345]	0.338* [0.331, 0.345]	0.395* [0.388, 0.402]
	NRI overall	0.752* [0.716, 0.788]	0.736* [0.700, 0.772]	0.732* [0.695, 0.769]
**Fatty liver disease**	IDI	0.013* [0.011, 0.015]	0.013* [0.011, 0.015]	0.020* [0.017, 0.023]
	NRI events	0.302* [0.244, 0.359]	0.388* [0.332, 0.444]	0.235* [0.176, 0.294]
	NRI non-events	0.341* [0.334, 0.349]	0.329* [0.321, 0.336]	0.312* [0.305, 0.319]
	NRI overall	0.643* [0.585, 0.701]	0.717* [0.660, 0.773]	0.547* [0.488, 0.606]
**Alcoholic liver disease**	IDI	0.019* [0.013, 0.025]	0.019* [0.013, 0.026]	0.071* [0.048, 0.094]
	NRI events	0.497* [0.362, 0.632]	0.371* [0.227, 0.515]	0.648* [0.529, 0.766]
	NRI non-events	0.603* [0.597, 0.609]	0.615* [0.609, 0.621]	0.726* [0.721, 0.731]
	NRI overall	1.100* [0.964, 1.235]	0.986* [0.841, 1.130]	1.374* [1.255, 1.492]
**Liver cirrhosis**	IDI	0.009* [0.004, 0.014]	0.009* [0.005, 0.014]	0.094* [0.069, 0.120]
	NRI events	0.301* [0.186, 0.415]	0.331* [0.217, 0.444]	0.466* [0.360, 0.572]
	NRI non-events	0.323* [0.316, 0.330]	0.335* [0.328, 0.343]	0.641* [0.635, 0.647]
	NRI overall	0.624* [0.509, 0.739]	0.666* [0.553, 0.780]	1.107* [1.001, 1.214]
**Liver failure**	IDI	0.007* [0.003, 0.011]	0.005* [0.002, 0.008]	0.035* [0.013, 0.057]
	NRI events	0.154 [-0.036, 0.344]	0.096 [-0.095, 0.287]	0.096 [-0.095, 0.287]
	NRI non-events	0.529* [0.522, 0.535]	0.400* [0.393, 0.406]	0.539* [0.533, 0.546]
	NRI overall	0.682* [0.492, 0.872]	0.496* [0.304, 0.687]	0.636* [0.444, 0.827]

IDI = integrated discrimination index , NRI = net reclassification improvement. An asterisk (*) indicates where the 95% confidence interval for the index does not contain zero. For all indices, a positive value indicates improvement in discrimination provided by the newly-developed risk score compared with the existing risk score. Statistics are calculated with the improveProb() function from the Hmisc r package.

Supplementary Figure 4: Hazard ratios for Remote model coefficients

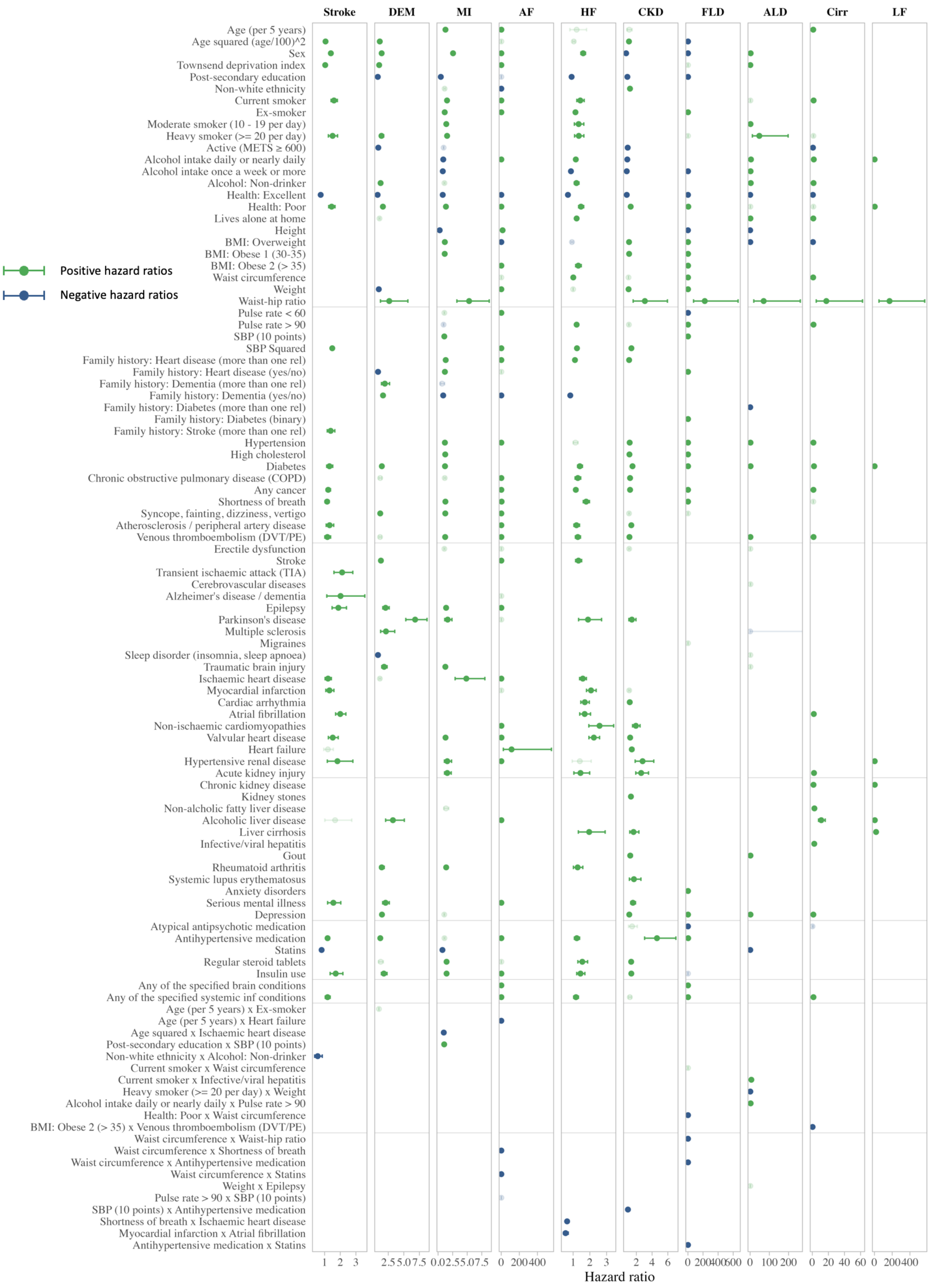

Model coefficients that are not significant after multiple testing adjustment (false discovery rate of 5%) has been applied are shown in greyed out colour. Please see Supplementary Spreadsheets 1 and 2 for more beta coefficients and detailed hazard ratio results

Supplementary Figure 5: Hazard ratios for Standard model coefficients

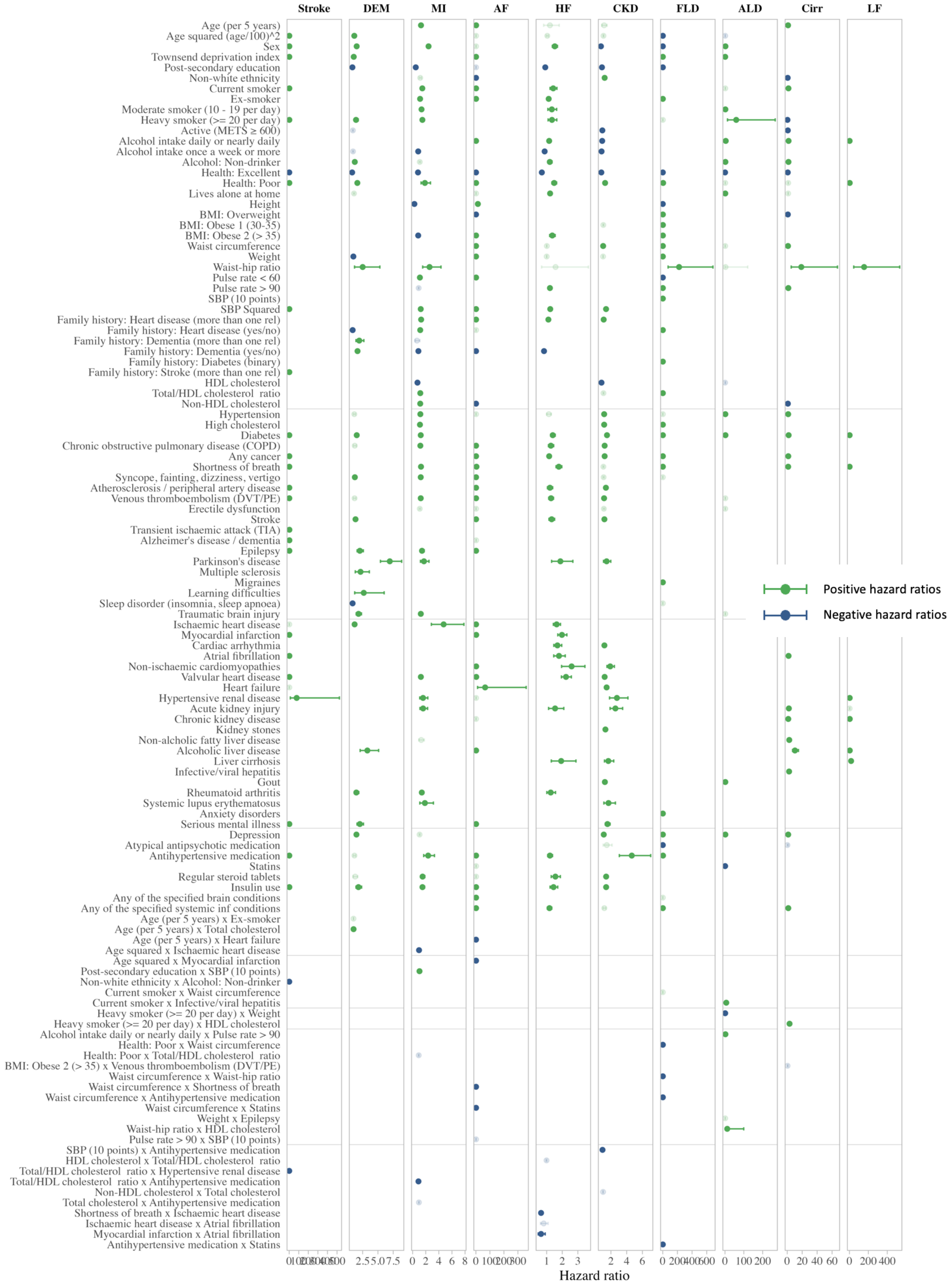

Model coefficients that are not significant after multiple testing adjustment (false discovery rate of 5%) has been applied are shown in greyed out colour. Please see Supplementary Spreadsheets 1 and 2 for more beta coefficients and detailed hazard ratio results

Supplementary Figure 6: Hazard ratios for Extended model coefficients

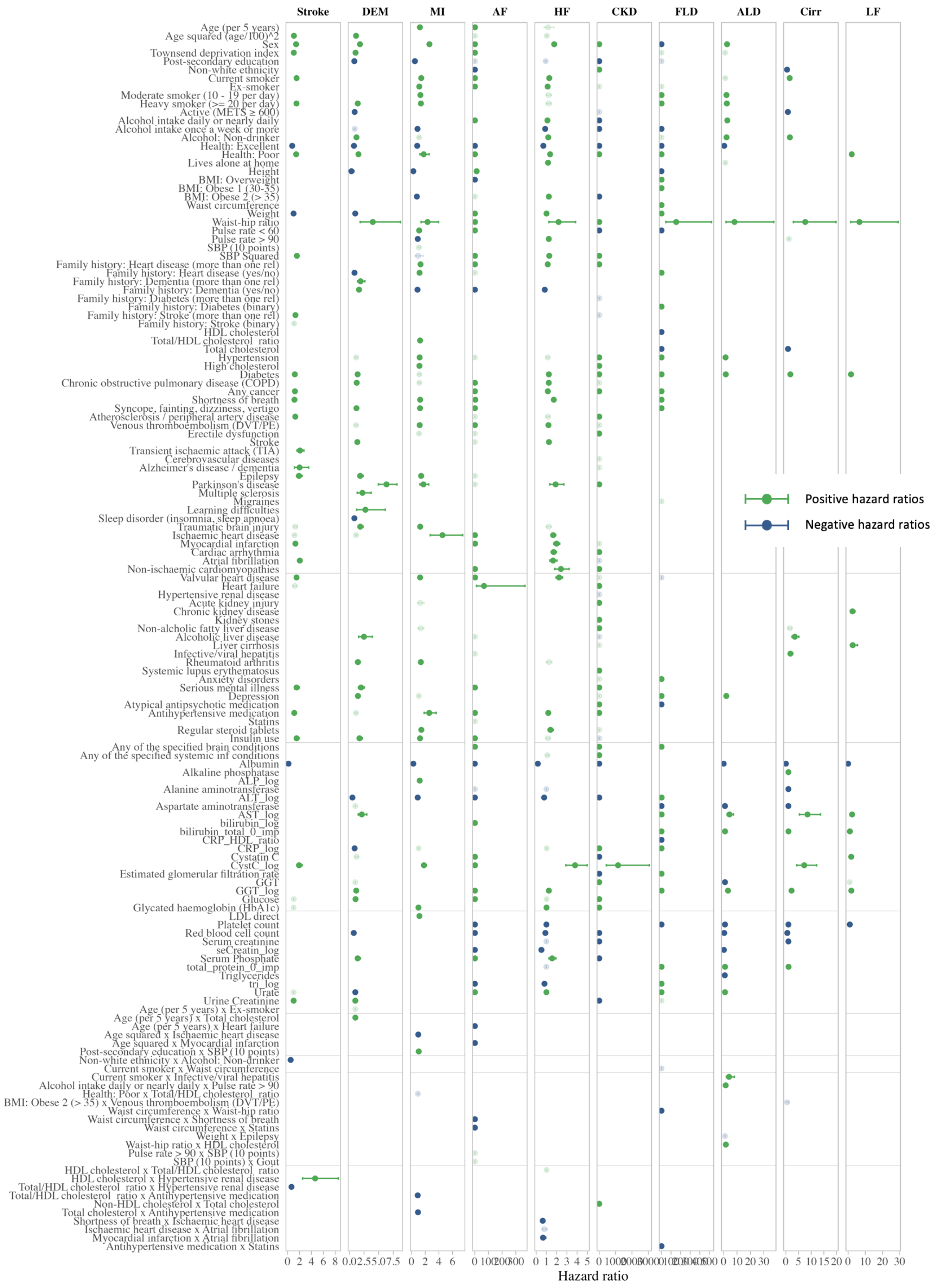

Model coefficients that are not significant after multiple testing adjustment (false discovery rate of 5%) has been applied are shown in greyed out colour. Please see Supplementary Spreadsheets 1 and 2 for more beta coefficients and detailed hazard ratio results
